# Valve replacement during pregnancy: literature review including new data from the Registry Of Pregnancy And Cardiac disease III

**DOI:** 10.1093/ejcts/ezaf180

**Published:** 2025-06-20

**Authors:** Johanna A van der Zande, Sergii Siromakha, Puck N J Peters, Ghada Youssef, Laura Galian-Gay, Magalie Ladouceur, Gretchen Wells, Johanna J M Takkenberg, Kevin M Veen, Karishma P Ramlakhan, Roger Hall, Mark R Johnson, Jolien W Roos-Hesselink, ROPAC Investigators, R Ferrari, A Alonso, J Bax, C Blomström-Lundqvist, S Gielen, P Lancellotti, A P Maggioni, N Maniadakis, F Pinto, F Ruschitzka, L Tavazzi, P Vardas, F Weidinger, U Zeymer, A Budaj, N Dagres, N Danchin, V Delgado, J Emberson, O Friberg, C P Gale, G Heyndrickx, B Iung, S James, A P Kappetein, A P Maggioni, N Maniadakis, K V Nagy, G Parati, A-S Petronio, M Pietila, E Prescott, F Ruschitzka, F Van de Werf, F Weidinger, U Zeymer, C P Gale, B Beleslin, A Budaj, O Chioncel, N Dagres, N Danchin, J Emberson, D Erlinge, M Glikson, A Gray, M Kayikcioglu, A P Maggioni, K V Nagy, A Nedoshivin, A-P Petronio, J W Roos-Hesselink, L Wallentin, U Zeymer, D Adlam, A L P Caforio, D Capodanno, M Dweck, D Erlinge, M Glikson, J Hausleiter, B Iung, M Kayikcioglu, P Ludman, L Lund, A P Maggioni, S Matskeplishvili, B Meder, K V Nagy, A Nedoshivin, D Neglia, A A Pasquet, J W Roos-Hesselink, F J Rossello, S M Shaheen, A Torbica, Jolien Roos-Hesselink, Roger Hall, William Parsonage, Werner Budts, Julie de Backer, Jasmin Grewal, Ariane Marelli, Guillaume Jondeau, Mark Johnson, Catherine Otto, Karen Sliwa, Aldo Maggioni, K Vardanyan, A Melkonyan, H Lachikyan, K Hakobyan, M Mazmanian, Yerevan: H Hayrapetyan, A Tavaracyan, H Poghosyan, R Hovhannisyan, S Sahakyan, S Martirosyan, J Harris, A Pasquet, M Morissens, T Besse-Hammer, B Dumoulin, J De Backer, L Campens, L Demulier, M De Hosson, W Budts, A Van de Bruaene, A Rampelberg, E Troost, L Roggen, P De Meester, J C Mwita, E Tefera, L Kontle, A Marelli, I Malhamé, J Grewal, M Janzen, P A Román Rubio, R Vasallo Peraza, G Vázquez Hernández, J E Pérez Torga, Y Gil Jiménez, M Meluzá Martín, R Almaleh, G Youssef, K Sorour, S Abebe, D Mekonnen, C Fekadu, D Yadeta, S Dupuis-Girod, L Delagrange, M Richardson, L Ghesquiere, O Domanski, M Gonzalez Estevez, Y Ould Hamoud, S Gautier, L Marsili, L Bal-Theoleyre, S Palazzolo, M Ladouceur, G Jondeau, A Bourgeois Moine, L Eliaou, O Milleron, M Tchitchinadze, Y Dulac, C Karsenty, N Souletie, F Bajanca, C Rickers, S Blankenberg, C Sinning, C Magnussen, E Zengin, G Mueller, R Schnabel, Y Von Kodolitsch, R Kozlik-Feldmann, H Baumgartner, R Schmidt, A Hellige, A Rietkötter, M Spartalis, A A Frogoudaki, A Arvanitaki, A Baroutidou, G Giannakoulas, C Karvounis, J P Gnanaraj, A P Steaphen, T Ethirajan, K Kannan, V Subramanian, A Surendran, J Gnanasekaran, V Natarajalingam, S Balasubramani, H Ali Farhan, I F Yaseen, E Mariucci, C Ciuca, F Marchi, G Benedetti, M Baroni, P Festa, A Parlanti, G Scognamiglio, F Fusco, B Sarubbi, M Merlo, B D'Agata Mottolese, C Carriere, G Sinagra, M Bobbo, F Ramani, F M Comoglio, R Bordese, A Pagano, N Montali, V Donvito, C A Remolif, F Petey, B Bouma, S Chamuleau, D Robbers-Visser, T Konings, H Dronkert, D Segers, R Van Kimmenade, H Van, Der Zwaan, G Tjalling Sieswerda, A Evers, T Schaap, K Bano, H Yasmeen, K Amir, N Patel, P Akhter, R Khan, A Shakeel, S Mahar, S Habib, M Lelonek, P Hoffman, M Lipczynska, L De Sousa, V Ferreira, T Mano, M Selas, R Cruz Ferreira, E Shlyakhto, O Irtyuga, G Sefieva, K Malikov, T Pervunina, U Shadrina, A Kinsara, D Galzerano, H Al Sergani, W Kurdi, N Kholaif, O Vriz, A Alhamshari, A Alsaigh, O Ahmad, S Alzaher, B Alamro, K Sliwa, F Jakoet-Bassier, L Galian-Gay, A Pijuan-Domenech, B Miranda-Barrio, B Gordon, E Furenäs, J Hlebowicz, F Wedlund, E Nagy, E Mattsson, M Majczuk Sennstrom, P Sörensson, C Christersson, A Lutvica, B Jönelid, T Achter, K Junus, H Gärdesten Wall, M Andreasson, D Tobler, J Bouchardy, F Brand, C Blanche, J Bouchardy, T Rutz, F Brand, U Canpolat, Y Z Sener, N Ozer, E Ayduk Gövdeli, Z Bugra, B Umman, P Karaca Özer, D Baykiz, D Mutlu, H Yalman, B Kilickiran Avci, S Catirli Enar, O Batukan Esen, D Oksen, V Lazoryshynets, S Siromakha, Y Davydova, A Limanska, I Zinovchyk, V Kravchenko, B Kravchuk, O Kravets, N Volkova, O Mazur, O Beregovyi, B T Salih, W A R Al Mahmeed, S Wani, F S Mohamed Farook, G Al Mansoori, S Prakash, R Afifi, D Milewicz, A Cecchi, G Wells, D Sparks, W Wagner, C Bigelow, L Colicchia, T Jentink, M Loichinger, R Saxena, W Wunderlich, C Longtin, P Klopper, R Gobar, J Chou, K Campbell, R Elder, D Halpern, A Hausvater, H Reynolds, N Bhalla, A Small, J Feinberg, P Panday, J Awerbach, J Porche, S Stack, L Mcgrath, A Khan, E Pare, P Woods, C Broberg, K Gibbins, K Brookfield, M Al-Sadawi, A Cove, N Mann

**Affiliations:** Department of Cardiology, Erasmus MC, University Medical Center Rotterdam, Rotterdam, The Netherlands; Department of Obstetrics and Gynecology, Erasmus MC—Sophia Children’s Hospital, University Medical Center Rotterdam, Rotterdam, The Netherlands; Department of Obstetric Cardiology, Amosov National Institute of Cardiovascular Surgery, Kyiv, Ukraine; Department of Cardiology, Erasmus MC, University Medical Center Rotterdam, Rotterdam, The Netherlands; Department of Cardiology, Cairo University Hospital, Cairo, Egypt; Department of Cardiology, University Hospital Vall d’Hebron, CIBER-CV, Barcelona, Spain; Department of Cardiology, Hôspital Européen Georges Pompidou, Paris, France; Department of Cardiology, Gill Heart and Vascular Institute, University of Kentucky, Lexington, Kentucky, USA; Department of Cardiothoracic Surgery, Erasmus MC, University Medical Center Rotterdam, Rotterdam, The Netherlands; Department of Cardiothoracic Surgery, Erasmus MC, University Medical Center Rotterdam, Rotterdam, The Netherlands; Department of Cardiology, Erasmus MC, University Medical Center Rotterdam, Rotterdam, The Netherlands; Department of Obstetrics and Gynecology, Erasmus MC—Sophia Children’s Hospital, University Medical Center Rotterdam, Rotterdam, The Netherlands; Department of Cardiology, University of East Anglia, Norwich, UK; Department of Obstetric Medicine, Imperial College London, Kensington, London, UK; Department of Cardiology, Erasmus MC, University Medical Center Rotterdam, Rotterdam, The Netherlands

**Keywords:** Valve replacement, Pregnancy, Cardiopulmonary bypass, Valve thrombosis

## Abstract

**OBJECTIVES:**

Heart valve replacement during pregnancy is sometimes unavoidable, and the need for anticoagulation further complicates these procedures. Our study describes cases of valve replacement in pregnancy enrolled in the Registry Of Pregnancy And Cardiac disease (ROPAC) III and gives an overview of the published literature.

**METHODS:**

We performed a systematic review with new data from the ROPAC III and data available in the literature. ROPAC III is a global, prospective, observational registry that included pregnant women with 1 or more prosthetic valves between January 2018 and April 2023. Electronic databases were searched for studies enrolling pregnant women who underwent valve replacement during pregnancy with a fetus *in utero*. The primary outcomes were maternal and fetal death. Mixed-effect logistic regression models were used to identify predictors for maternal and fetal mortality.

**RESULTS:**

A valve replacement was performed in 11 pregnancies. The mother and fetus died in 1 case, and in 2 cases, reversible postoperative complications occurred. We found 74 cases in the literature and calculated an overall maternal and fetal death rate of 9% and 34%, respectively. All maternal deaths occurred in women with a replacement of a prosthetic valve in mitral position. We found valve replacement in the 1st trimester (OR 10.0) and acute malfunctioning of an existing prosthetic valve (OR 19.7) as predictors for maternal mortality, and replacement of an existing prosthetic valve (OR 4.8) as predictor for fetal mortality.

**CONCLUSIONS:**

Valve replacement during pregnancy carries a high maternal and fetal death, especially in women who need a replacement of an existing prosthetic valve.

## Graphical Abstract

**Figure:**
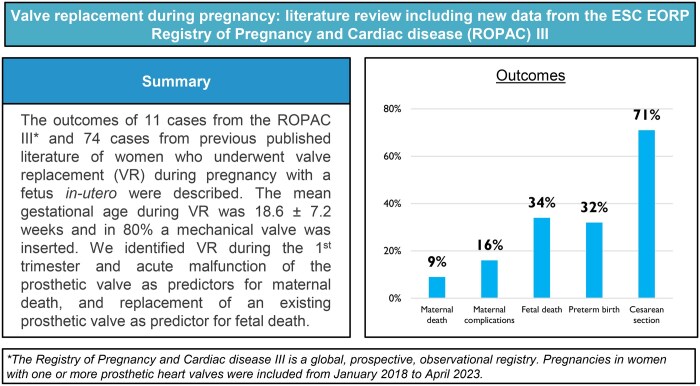

## INTRODUCTION

Pregnancy in women with cardiac disease poses an elevated risk of maternal and fetal morbidity and mortality, especially to those who are not able to sufficiently adapt to the haemodynamic challenges of pregnancy [1, 2]. However, advances in cardiac surgery and obstetric care have resulted in improved maternal outcomes [3]. When complications do arise, cardiac surgery may be the only option to save a mother’s life. Currently, maternal mortality during cardiac surgery on cardiopulmonary bypass (CPB) is similar to that of non-pregnant women [4], and the risk of fetal mortality is very high (27%) with CPB and abnormally prolonged and forceful uterine contractions during cardiac surgery being responsible [5]. The option of delaying surgery until the fetus is sufficiently mature is not always available due to the urgency of the clinical situation. Women with a mechanical heart valve are at risk for thromboembolic and haemorrhagic events during pregnancy. They have to receive adequate anticoagulation to avoid valve thrombosis, but over-anticoagulation is associated with an increased haemorrhage risk. Prosthetic valve thrombosis is the most feared complication with an incidence around 5% [6], and definitive treatment either in the form of thrombolysis or valve replacement is usually unavoidable. Our study aims to give an overview of the background and underlying diagnoses of women who underwent valve replacement during pregnancy with fetus *in utero*. We describe new cases from the Registry Of Pregnancy And Cardiac disease (ROPAC) III and then present a review of all the cases in the literature. Our hypothesis is that valve replacement during pregnancy is associated with a high risk of maternal and fetal death.

## MATERIALS AND METHODS

### Registry Of Pregnancy And Cardiac disease

Due to the need of data on policy and pregnancy outcomes in women with aortopathy and prosthetic valves, ROPAC III was introduced to collect this data throughout the world. Pregnant women were prospectively included, after written informed consent, from 2018 until April 2023. In this study, we describe cases from the ROPAC III in which a valve replacement was performed during pregnancy with fetus *in utero*.

### Literature review

As surgical valve replacement with the fetus *in utero* is uncommon and the body of literature small, we decided to collect published cases to increase numbers enabling more robust data. The primary outcomes were maternal and fetal death. The following databases were searched from their inception until 15 September 2023: Medline, Embase and Cochrane Central Register of Controlled trials. The full search strategy and methods of screening and data collection are described in Supplementary Material, Table S1.

### Statistical analysis

Statistical analysis was performed using SPSS IBM statistics version 25.0 and R version 4.0.5. Continuous variables were expressed as mean with standard deviation (SD), or as median with interquartile range (IQR) if skewed. Categorical data were expressed as number with percentage, and pairwise deletion was used for the calculation of these percentages. Baseline and outcome data were stratified by trimester and by year of publication (<2010 and ≥2010) to evaluate changes over time. The 1st trimester was defined as gestational age of 1–14 weeks, 2nd trimester as gestational age of 15–27 weeks and 3rd trimester as gestational age >27 weeks. To analyse statistical significance according to trimester the chi-squared test (categorical data), Fisher-Freeman-Halton exact test (categorical data with an expected count <5) or analysis of variance (continuous data) was used. We then compared surgery characteristics and outcomes between the cases with and without maternal death, between the cases with and without fetal death, and between low-or-middle-income countries (LMIC) and high-income countries (HIC; based upon The International Monetary Fund classification) [7].

To investigate predictors for maternal and fetal mortality, we used mixed-effect logistic regression models with centre as random effect and the covariate of interest as fixed effect to account for heterogeneity among centres. For maternal and fetal mortality, univariable models were constructed. Only variables with <25% missing data were included in these mixed-effect logistic regression models. A *P-*value of <0.05 was considered statistically significant.

## RESULTS

### Registry Of Pregnancy And Cardiac disease

In 11 (1.0%) out of 1105 pregnancies enrolled in the ROPAC III, the woman underwent valve replacement during their pregnancy with a fetus *in utero*. The median maternal age was 27 (24–33) years (Table 1). Twelve valves were inserted, of which 11 were mechanical valves and 1 was a tissue valve. The indications for valve replacement were native valve stenosis and/or regurgitation in the context of congenital heart disease (*n* = 4), valve thrombosis (*n* = 4), rheumatic heart disease (*n* = 2) or unknown aetiology (*n* = 1). The median gestational age during valve replacement was 20 (13–22) weeks. The median bypass time and aortic clamp time was 124 (67–131) and 92 (54–109) min, respectively (Supplementary Material, Table S2). In 3 (27.3%) cases, postoperative complications occurred: 1 woman went into cardiogenic shock which resulted in maternal and fetal mortality, 1 woman developed a complete AV block requiring pacemaker implantation, and 1 woman had an alveolar haemorrhage (Table 1). One woman experienced minor vaginal bleeding episodes during the remainder of the pregnancy. The median gestational age at delivery was 38 (27–40) weeks and 2 (20.0%) women delivered preterm (at 27 and 36 weeks of gestation). Of the 10 women with an ongoing pregnancy after valve replacement, 8 were delivered by Caesarean section. Postpartum haemorrhage requiring intervention occurred in 2 cases. There was 1 neonatal death after emergency Caesarean section due to fetal distress with hydrocephalus. Small for gestational age was reported in 2 cases and in 1 infant cerebral palsy was diagnosed at 6 months of age. Details on anticoagulation and anti-Xa level target, if applicable, are presented in Supplementary Material, Table S3.

**Table 1:** Valve replacement during pregnancy, details of ROPAC III cases

	Valvular diagnosis	Additional diagnosis	Maternal age (years)	Gestational age during valve replacement (weeks)	Position of the prosthetic valve(s)	Postoperative complications	Gestational age at delivery (weeks^+days^)	Mode of delivery	Peripartum complications	Neonatal complications	Additional information
First prosthetic valve during pregnancy											
1.	Aortic regurgitation + Mitral regurgitation and stenosis	Rheumatic heart disease	Unknown	4	Aortic position (mech) + Mitral position (mech)	None	38^+0^	Caesarean section (planned)	None	None	Pregnancy unknown at time of valve replacement
2.	Aortic stenosis	Unicuspid aortic valve + heart failure	23	20	Aortic position (mech)	None	36^+0^	Caesarean section (emergency, fetal distress)	None	Neonatal death, child with hydrocephalus	–
3.	Aortic stenosis (PG 102 mmHg)	BAV + pulmonary hypertension + heart failure	30	20	Aortic position (mech)	None	38^+0^	Caesarean section (planned)	None	None	–
4.	Aortic stenosis (PG 101 mmHg)	Unicuspid aortic valve + pulmonary hypertension+ ascending aortic dilatation (40 mm)	22	22	Aortic position (mech)	Complete AV block > Pacemaker implantation	39^+0^	Caesarean section (planned)	None	None	Cerebral palsy detected after 6 months of follow-up
5.	Mitral stenosis (valve area 13 mm^2^) and regurgitation (PG 30 mmHg)	Pulmonary hypertension	27	23	Mitral position (mech)	None	37^+6^	Caesarean section (planned)	None	Small for gestational age (birth weight <10th percentile)	–
6.	Aortic stenosis (PG 45 mmHg)	BAV + ascending aortic dilation (42 mm) with 6 mm growth during pregnancy + heart failure	26	24	Aortic position (mech)	None	39^+0^	Caesarean section (planned)	None	None	Surgical repair of aortic dilatation during same procedure
7.	Mitral stenosis (valve area 11 mm^2^) and regurgitation	Rheumatic heart disease + Pulmonary hypertension	25	20	Mitralposition (bio) 27 mm	None	37^+1^	Caesarean section (planned)	None	None	–
Replacement of prosthetic valve during pregnancy											
8.	2x AVR (mech), MVR (mech)	Valve thrombosis of the prosthetic mitral valve (PTT 50 s, mean gradient 24 mmHg)	Unknown	7	Mitral position (mech)	Cardiogenic shock	–	–	–	Fetal death	Maternal mortality 2 days after valve replacement
9.	MVR (mech)	Valve thrombosis of the prosthetic mitral valve + atrial flutter	35	14	Mitral position (mech)	Alveolar haemorrhage	39^+5^	Assisted vaginal delivery (forceps)	Postpartum haemorrhage solved with vaginal tamponade	Small for gestational age (birth weight <10th percentile)	–
10.	AVR (mech), MVR (mech)	Valve thrombosis of the prosthetic mitral valve (anti-Xa 0.9 U/ml)	28	19	Mitral position (mech)	None	37^+4^	Vaginal delivery	None	None	Episodes of minor vaginal bleeding during pregnancy
11.	MVR (mech)	Valve thrombosis of the prosthetic mitral valve	35	13	Mitral position (mech)	Atrial fibrillation	27^+2^	Caesarean section (emergency, fetal distress)	Incisional haematoma > reoperation	None	–

AV: atrioventricular; AVR: aortic valve replacement; BAV: bicuspid aortic valve; bio: biological valve; mech: mechanical valve; MVR: mitral valve replacement; PG: peak gradient; PTT: prothrombin time.

### Literature review

**Figure 1:**
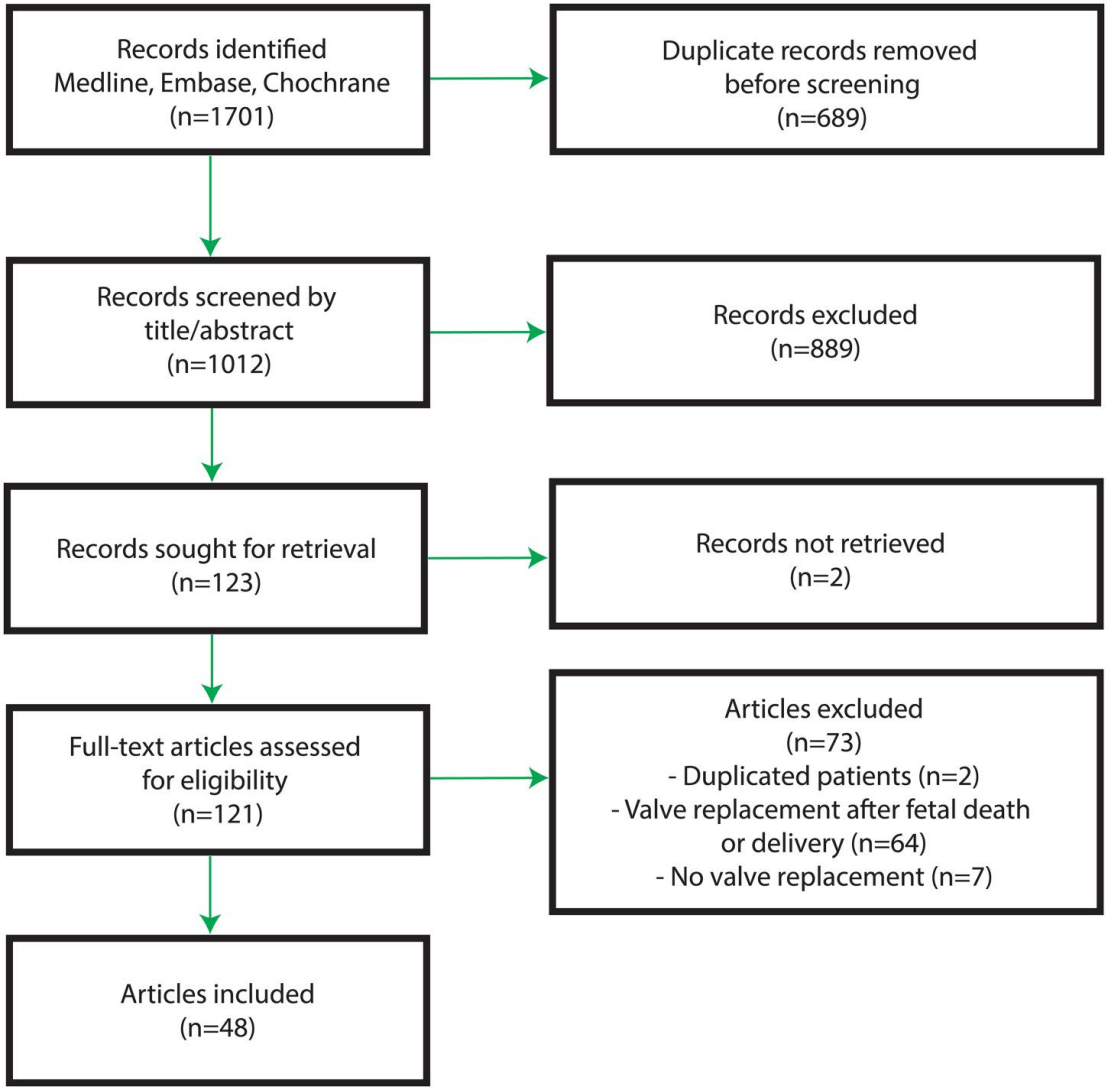
Flowchart of literature search and selection process.

On 25th of September 2023, 1701 articles were identified by our database search, of which 1012 were potentially relevant unique articles and screened based on titles and abstracts (Fig. 1). Full-text screening was subsequently performed on 121 records, of which 48 studies were finally included (Supplementary Material). In these 48 studies, 74 cases of women who underwent surgical valve replacement while pregnant were described and characteristics were collected (Supplementary Material, Tables S4 and S5). The extent of missing data in these studies are presented in Supplementary Material, Table S6.

### Characteristics of current valve replacement surgery

The aggregated surgery characteristics of the total population, including ROPAC III data and the data from the literature, are presented in Table 2. The median maternal age was 27 (24–33) years, and the mean gestational age at time of valve surgery was 18.6 ± 7.2 weeks. Most of the women underwent surgery during the 2nd trimester (51.8%, *n* = 44). Left heart valve replacement was performed most frequently: 65.9% (*n* = 56) underwent mitral valve replacement, 25.9% (*n* = 22) underwent aortic valve replacement, and 5.9% (*n* = 5) underwent both aortic and mitral valve replacement. In 2.4% (*n* = 2) cases tricuspid valve replacement was performed. In most of the cases, a mechanical valve was chosen (80.2%), especially in the cases published ≥2010 (87.7%). In 79.3% of the women operated in the 1st trimester, the surgery involved the re-replacement of a prosthetic valve, which is significantly higher than in the women operated in the 2nd trimester (40.9%; *P* = 0.002) but not different compared to the 3rd trimester (54.5%; *P* = 0.137). The mean CPB-time and cross-clamp time was 100 ± 35 min and 65 ± 28 min, respectively, and differed between the trimesters (*P* = 0.037 and *P* = 0.024, respectively). No differences were found in surgery characteristics between cases published <2010 and ≥2010. Fetal monitoring during the valve replacement was performed in 27 (31.5%) cases with differences among trimester (*P* = 0.016) and year of publication (*P* = 0.044).

**Table 2:** Surgery characteristics of the total population (ROPAC III and literature cases) and stratified by trimester and year of publication

	Total (*n* = 85)	1st trimester (*n* = 29)	2nd trimester (*n* = 44)	3rd trimester (*n* = 11)	*P*-value	Published <2010 (*n* = 27)	Published ≥2010 (*n* = 58)	*P*-value
Maternal age during pregnancy, median (years)	27 (24–33)	29 (25–34)	27 (24–34)	24 (20–27)	**0.034** ^a^ ^,^ ^b^	25 (23–31)	28 (24–34)	0.382
Gestational age during valve replacement (weeks)	18.6 ± 7.2	10.9 ± 2.9	20.7 ± 3.3	30.6 ± 1.6	**<0.001** ^a,b,c^	20.8 ± 8.6	17.6 ± 6.2	0.052
Indication for valve replacement					**0.002**			0.248
Valve thrombosis	21 (24.7)	13 (44.8)	5 (11.4)	3 (27.3)	**0.004**	7 (25.9)	14 (24.1)	1.000
Stuck PV/acute malfunction PV^d^	26 (30.6)	10 (34.5)	13 (29.5)	3 (27.3)	0.946	5 (18.5)	22 (37.9)	0.085
Endocarditis	10 (11.8)	0 (0)	10 (22.7)	0 (0)	**0.005**	5 (18.5)	5 (8.6)	0.277
Native valve disease^e^	27 (31.8)	6 (20.7)	16 (36.4)	5 (45.5)	0.240	10 (37.0)	17 (29.3)	0.617
Type of valve								
Mechanical valve^d^	65 (80.2)	24 (82.8)	34 (79.1)	6 (75.0)	0.849	15 (62.5)	50 (87.7)	**0.014**
Tissue valve	16 (19.8)	5 (17.2)	9 (20.9)	2 (25.0)	0.849	9 (37.5)	7 (12.3)	**0.014**
Position								
Aortic	22 (25.9)	5 (17.2)	15 (34.1)	2 (18.2)	0.252	12 (44.4)	10 (17.2)	**0.015**
Mitral^d^	56 (65.9)	22 (75.9)	26 (59.1)	7 (63.6)	0.347	11 (40.7)	45 (77.6)	0.001
Aortic + Mitral	5 (5.9)	1 (3.4)	2 (4.5)	2 (18.2)	0.255	3 (11.1)	2 (3.4)	0.321
Tricuspid	2 (2.4)	1 (3.4)	1 (2.3)	0 (0)	1.000	1 (3.7)	1 (1.7)	0.537
Re-replacement^d^	48 (56.5)	23 (79.3)	18 (40.9)	6 (54.5)	**0.005** ^c^	12 (44.4)	36 (62.1)	0.161
CPB duration (min)	100 ± 35	79 ± 36	109 ± 29	78 ± 49	**0.037**	98 ± 39	102 ± 33	0.755
Cross-clamp time (min)	65 ± 28	48 ± 31	72 ± 26	35 ± 3	**0.024** ^b^	64 ± 29	65 ± 28	0.862
Hypothermia	26 (68.4)	8 (88.9)	15 (62.5)	3 (60.0)	0.405	15 (75.0)	11 (61.1)	0.489
Pulsatile flow	20 (74.1)	4 (80.0)	15 (78.9)	1 (33.3)	0.297	8 (66.7)	12 (80.0)	0.662
Pump flow (l/min/m^2^)	2.8 ± 0.5	2.6 ± 0.2	2.8 ± 0.6	3.0 ± 0.8	0.691	2.7 ± 0.4	2.8 ± 0.6	0.848
Mean arterial pressure (mmHg)	68 ± 6	64 ± 8	69 ± 5	70 ± 9	0.312	68 ± 7	68 ± 6	0.692
Fetal monitoring during surgery	27 (31.8)	4 (13.8)	17 (38.6)	6 (54.5)	**0.016** ^a^ ^,^ ^c^	13 (48.1)	14 (24.1)	**0.044**

Data are presented as *n* (%) or mean ± SD unless otherwise specified. Percentages are calculated using pairwise deletion. Bold values indicate p<0.05.

a Significant difference 1st trimester vs 3rd trimester.

b Significant difference 2nd trimester vs 3rd trimester.

c Significant difference 1st trimester vs 2nd trimester.

d Trimester unknown during surgery in 1 patient.

e Aortic stenosis and/or regurgitation or mitral stenosis and/or regurgitation in the context of rheumatic heart disease (*n* = 12), congenital heart disease (*n* = 4) or unknown aetiology (*n* = 11).

CPB: cardiopulmonary bypass; PV: prosthetic valve.

### Maternal mortality and complications

Maternal mortality occurred in 8 (9.4%) cases (Table 3). Compared to the 2nd trimester, maternal mortality was more common when the operation occurred in the 1st trimester (20.7% vs 2.3%; *P* = 0.029). Maternal (post-operative) complications, excluding maternal death, were described in another 12 (15.6%) cases: 1 woman had anoxic encephalopathy due to a cardiac arrest during surgery, 3 women had a haemorrhagic event, 3 women had a thromboembolic event, 3 women developed arrhythmia, 2 of whom required a pacemaker implantation, and another woman had pericardial effusion which was resolved after corticosteroids. The surgery characteristics and outcomes compared between the cases with and without maternal death are presented in Table 4. All maternal mortality cases involved a re-replacement of the mitral valve. Fetal death was more common when the mother died compared to when the mother survived (87.5% vs 28.6%, *P* = 0.002). There were no differences in surgery characteristics between the cases with and without maternal death. We identified valve replacement during the 1st trimester (OR 10.0; 95% CI 1.0–98.1) and a stuck or acute malfunction of the prosthetic valve (OR 19.7; 95% CI 2.3–170.2) as predictors for maternal death (Table 5).

**Table 3:** Outcomes of the total population (ROPAC III and literature cases) and stratified by trimester and year of publication

	Total (*n* = 85)	1st trimester (*n* = 29)	2nd trimester (*n* = 44)	3rd trimester (*n *= 11)	*P*-value	Published <2010 (*n* = 27)	Published ≥2010 (*n* = 58)	*P*-value
Maternal death	8 (9.4)	6 (20.7)	1 (2.3)	1 (9.1)	**0.029** ^a^	1 (3.7)	7 (12.1)	0.426
Maternal complications^b^	12 (15.6)	5 (21.7)	7 (16.3)	0 (0)	0.369	7 (26.9)	5 (9.8)	0.093
Fetal death	29 (34.1)	13 (44.8)	13 (29.5)	3 (27.3)	0.404	5 (18.5)	24 (41.4)	0.050
Delivery^c^	54 (65.1)	14 (51.9)	31 (70.5)	8 (72.7)	0.258	20 (80.0)	34 (58.6)	0.080
Gestational age during delivery (weeks)	36.4 ± 3.1	36.9 ± 3.7	36.2 ± 3.1	36.6 ± 2.9	0.823	36.1 ± 3.5	36.7 ± 2.9	0.561
Preterm birth	13 (31.7)	2 (20.0)	8 (34.8)	3 (37.5)	0.731	6 (33.3)	7 (30.4)	1.000
Caesarean section^c^	37 (71.2)	9 (75.0)	22 (71.0)	5 (62.5)	0.827	11 (61.1)	26 (76.5)	0.337

Data are presented as *n* (%) or mean ± SD unless otherwise specified. Percentages are calculated using pairwise deletion. Bold values indicate p<0.05.

a Significant difference 1st trimester vs 2nd trimester.

b Maternal death cases excluded.

c Trimester unknown during surgery in 1 patient.

**Table 4:** Characteristics and outcomes in cases resulted in maternal death compared to maternal survival cases

	Total (*n* = 85)	Maternal death (*n* = 8)	No maternal death (*n* = 77)	*P*-value
Maternal age during pregnancy (years)	27 (24–33)	29.0 ± 7.4	27.8 ± 5.8	0.614
Gestational age during valve replacement (weeks)	18.6 ± 7.2	15.1 ± 8.1	19.0 ± 7.0	0.148
Indication for valve replacement				**0.006**
Valve thrombosis	21 (24.7)	1 (12.5)	20 (26.0)	0.673
Stuck PV/acute malfunction PV	26 (30.6)	7 (87.5)	20 (26.0)	**0.001**
Endocarditis	10 (11.8)	0 (0)	10 (13.0)	0.588
Native valve disease^a^	27 (31.8)	0 (0)	27 (35.1)	0.051
Type of valve				
Mechanical valve	65 (80.2)	8 (100.0)	57 (78.1)	0.346
Tissue valve	16 (19.8)	0 (0)	16 (21.9)	0.346
Position				
Aortic	22 (25.9)	0 (0)	22 (28.6)	0.105
Mitral	56 (65.9)	8 (100.0)	48 (62.3)	**0.047**
Aortic + Mitral	5 (5.9)	0 (0)	5 (6.5)	1.000
Tricuspid	2 (2.4)	0 (0)	2 (2.6)	1.000
Re-replacement	48 (56.5)	8 (100.0)	40 (51.9)	**0.009**
CPB duration (min)	100 ± 35	127 ± x	100 ± 35	0.449
Cross-clamp time (min)	65 ± 28	45 ± x	65 ± 28	0.484
Hypothermia	26 (68.4)	0 (0)	26 (68.4)	0.100
Pulsatile flow	20 (74.1)	1 (100.0)	19 (73.1)	1.000
Pump flow (l/min/m^2^)	2.8 ± 0.5	2.4 ± x	2.8 ± 0.6	0.504
Mean arterial pressure (mmHg)	68 ± 6	70 ± x	68 ± 7	0.755
Fetal monitoring during surgery	27 (31.8)	0 (0)	27 (35.1)	0.051
Fetal death	29 (34.1)	7 (87.5)	22 (28.6)	**0.002**

Data are presented as *n* (%) or mean ± SD unless otherwise specified. Percentages are calculated using pairwise deletion. Bold values indicate p<0.05.

a Aortic stenosis and/or regurgitation or mitral stenosis and/or regurgitation in the context of rheumatic heart disease (*n* = 12), congenital heart disease (*n* = 4) or unknown aetiology (*n* = 11).

CPB: cardiopulmonary bypass; PV: prosthetic valve.

**Table 5:** Predictors for maternal and fetal mortality. Mixed-effect logistic regression models with centre as random effect and the covariate of interest as fixes effect to account for heterogeneity among centres

	OR (95% CI)	*P*-value
**Predictors for maternal mortality** ^a^		
Maternal age	1.08 (0.91–1.26)	0.390
Trimester		
1st trimester^b^	9.97 (1.01–98.05)	**0.049**
3rd trimester^b^	5.26 (0.25–111.52)	0.286
Indication for valve replacement		
Valve thrombosis	0.64 (0.05–7.52)	0.722
Stuck PV/acute malfunction PV	19.71 (2.28–170.21)	**0.007**
**Predictors for fetal mortality**		
Maternal age	1.12 (0.98–1.28)	0.112
Trimester		
1st trimester^b^	3.21 (0.54–19.20)	0.201
3rd trimester^b^	1.59 (0.15–16.94)	0.700
Indication for valve replacement		
Valve thrombosis	5.43 (0.56–53.24)	0.146
Stuck PV/acute malfunction PV	2.59 (0.58–11.64)	0.215
Endocarditis	1.15 (0.15–8.97)	0.895
Native valve disease	0.14 (0.03–0.71)	**0.018**
Mechanical valve	0.71 (0.12–4.08)	0.697
Re-replacement	4.76 (1.23–18.44)	**0.024**
Fetal monitoring during surgery	0.36 (0.08–1.61)	0.180

a Not possible for endocarditis or native valve disease as indication for valve replacement, and for re-replacement and mechanical valve because of quasi complete separation.

b Compared to 2nd trimester.

Bold values indicate p<0.05. PV: prosthetic valve.

### Fetal death

Fetal death occurred in 29 (34.1%) cases, of which 4 were diagnosed during and 25 diagnosed after the surgery (Table 3). Table 6 describes the surgery characteristics and outcomes comparing cases with and without fetal death. Most of the fetal deaths were associated with surgery to replace malfunctioning prosthetic valves (79.3%). In our univariable mixed-effect logistic regression model, replacement of a prosthetic valve (OR 4.8; 95% CI 1.2–18.4) was a predictor for fetal death (Table 5).

**Table 6:** Characteristics and outcomes in cases resulted in fetal death compared to fetal survival cases

	Total (*n* = 85)	Fetal death (*n* = 29)	No fetal death (*n* = 56)	*P*-value
Maternal age during pregnancy (years)	27 (24–33)	29.3 ± 6.0	27.2 ± 5.9	0.123
Gestational age during valve replacement	18.6 ± 7.2	16.8 ± 7.1	19.6 ± 7.1	0.090
Indication for valve replacement				**0.005**
Valve thrombosis	21 (24.7)	8 (27.6)	13 (23.2)	0.792
Stuck PV/acute malfunction PV	26 (30.6)	15 (51.7)	12 (21.4)	**0.007**
Endocarditis	10 (11.8)	3 (10.3)	7 (12.5)	1.000
Native valve disease^a^	27 (31.8)	3 (10.3)	24 (42.9)	**0.003**
Type of valve				
Mechanical valve	65 (80.2)	23 (82.1)	42 (79.2)	0.781
Tissue valve	16 (19.8)	5 (17.9)	11 (20.8)	0.781
Position				
Aortic	22 (25.9)	6 (20.7)	16 (28.6)	0.453
Mitral	56 (65.9)	21 (72.4)	35 (62.5)	0.471
Aortic + Mitral	5 (5.9)	1 (3.4)	4 (7.1)	0.657
Tricuspid	2 (2.4)	1 (3.4)	1 (1.8)	1.000
Re-replacement	48 (56.5)	23 (79.3)	25 (44.6)	**0.003**
CPB duration (min)	100 ± 35	110 ± 29	97 ± 37	0.309
Cross-clamp time (min)	65 ± 28	56 ± 17	68 ± 31	0.295
Hypothermia	26 (68.4)	2 (50.0)	24 (70.6)	0.577
Pulsatile flow	20 (74.1)	8 (100.0)	12 (63.2)	0.068
Pump flow (l/min/m^2^)	2.8 ± 0.5	2.7 ± 0.5	2.8 ± 0.6	0.507
Mean arterial pressure (mmHg)	68 ± 6	71 ± 2	67 ± 7	0.140
Fetal monitoring during surgery	27 (31.8)	6 (20.7)	21 (37.5)	0.144
Maternal death	8 (9.4)	7 (24.1)	1 (1.8)	**0.002**
Maternal complications^b^	12 (15.6)	2 (9.1)	10 (18.2)	0.491

Data are presented as *n* (%) or mean ± SD unless otherwise specified. Percentages are calculated using pairwise deletion. Bold values indicate p<0.05.

a Aortic stenosis and/or regurgitation or mitral stenosis and/or regurgitation in the context of rheumatic heart disease (*n* = 12), congenital heart disease (*n* = 4) or unknown aetiology (*n* = 11).

b Maternal death cases excluded.

CPB: cardiopulmonary bypass; PV: prosthetic valve.

### Delivery

In 97.6% of cases with an ongoing pregnancy after the valve replacement, data on delivery were available (Table 3). The mean gestational age during delivery was 36.4 ± 3.1 weeks. Preterm birth occurred in 31.7%, and most of the women were delivered by Cesarean section (71.2%).

### LMIC versus HIC

**Figure 2:**
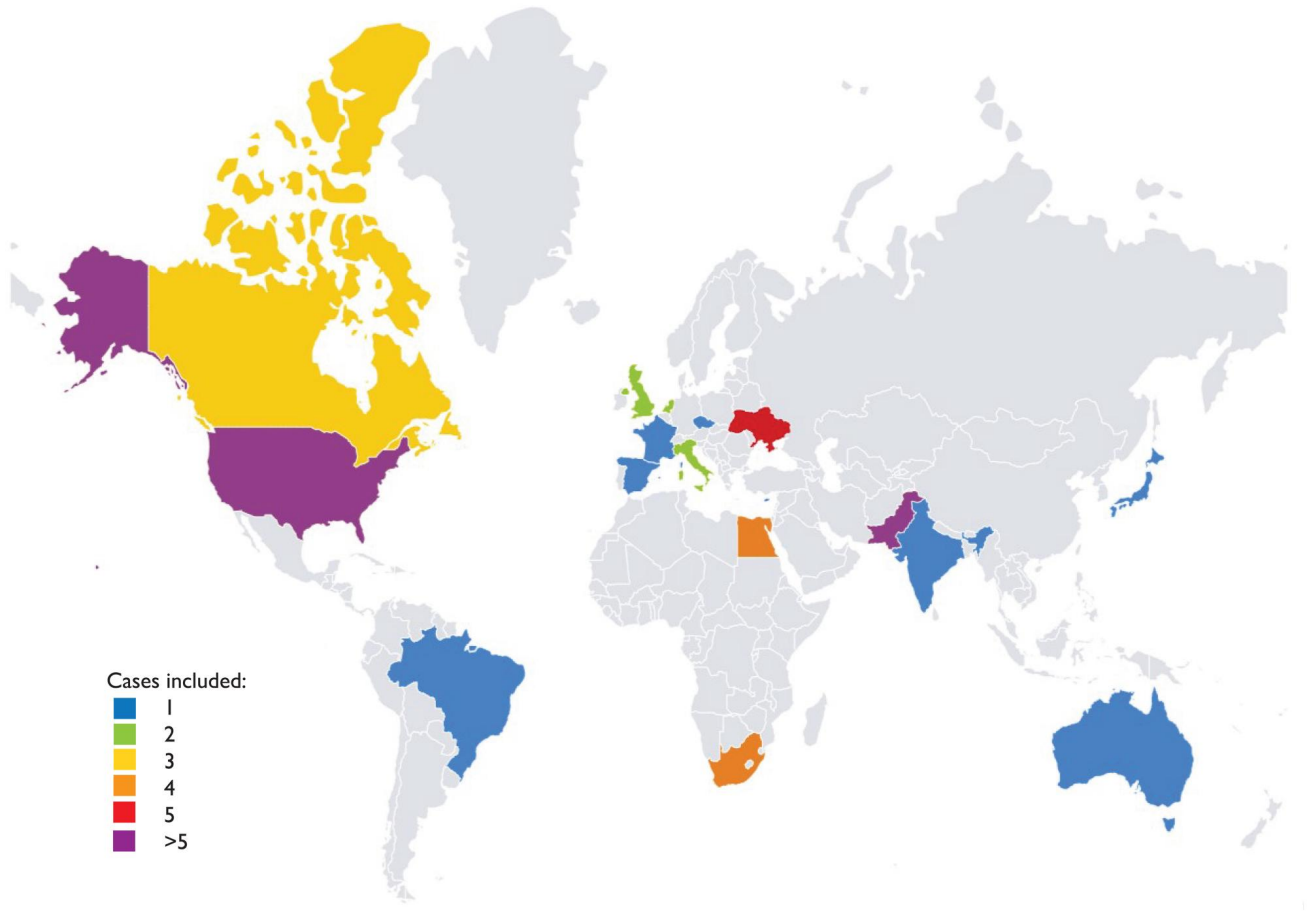
World map showing the origin of included cases, with countries colour-coded according to the number of cases included.

More than half of the cases were from a LMIC (55.3%) (Fig. 2) (Supplementary Material, Table S7). No differences were seen in maternal age during pregnancy and gestational age during valve replacement between cases from LMICs compared to cases from HICs. Differences in indication for valve replacement were observed (*P* < 0.001) with a stuck or acute malfunctioning prosthetic valve being more common in cases from LMICs (46.8% vs 13.2%, *P* = 0.001) and valve thrombosis and endocarditis being more common in HICs (14.9% vs 26.8%, *P* = 0.024 and 0% vs 26.3%, *P* < 0.001, respectively). Most of the prosthetic valves implanted in LMIC were mechanical valves (91.1%) in mitral position (76.6%). Fetal monitoring during valve replacement was less often performed in LMICs compared to HICs (19.1% vs 47.4%, *P* = 0.009). We found no differences in maternal and fetal death between LMICs and HICs; however, more maternal complications were reported in HICs (5.0% vs 27.0%, *P* = 0.011).

## DISCUSSION

This review on valve replacement during pregnancy with a fetus *in utero* includes prospective data from the ROPAC III and previous published case reports or case series. Overall, the maternal death rate was 9.4%, while the fetal death rate was 34.1%. We found ‘need for valve replacement in the 1st trimester’ and ‘a stuck or acute malfunction of the prosthetic valve’ as predictors for maternal death, and ‘replacement of an existing prosthetic valve’ as predictor for fetal death. All maternal mortalities occurred in women with a replacement of a prosthetic valve in mitral position.

### Maternal outcome

Maternal mortality was high emphasizing the importance of early detection of prosthetic valve malfunction resulting from valve thrombosis. The diagnosis is often difficult but should be considered in every pregnant woman with a mechanical valve, presenting with symptoms of shortness of breath. Women with a mechanical valve in mitral position are at particularly high risk and should be investigated carefully. The 1st trimester seems to be the most vulnerable period. This is in line with a recent patient-level meta-analysis of van Steenbergen *et al.* [8]. This meta-analysis included 266 cases of women who underwent any type of cardiac surgery with fetus *in utero* and not only valve replacement. They included, for example, women with an aortic dissection, which is known to be associated with a high maternal mortality risk [9] and to occur most commonly in the 3rd trimester. Although their calculated maternal mortality rate was not statistically different between trimesters, the maternal mortality rate in the 1st trimester was clinically higher than in 2nd and 3rd trimester (12.1% in 1st trimester, 4.6% in 2nd trimester, 8.8% in 3rd trimester) [8]. We suspect that the risk of maternal death does not depend solely on the trimester but is more likely related to the severity of the clinical situation and the indication for immediate intervention, as we also found a stuck or acute malfunction of a prosthetic valve as predictor for maternal death. All maternal deaths occurred in women who required valve replacement of an existing prosthetic valve, which may also be related to the complexity of a redo surgery, as it is well accepted that redo cardiac operations are associated with a high rate of morbidity and mortality [10]. Additionally, the follow-up in the included studies is generally short, but it is known that intensive care admission during pregnancy is associated with reduced physical and mental health, particularly for women who have lost their baby [11]. Postnatal care and psychological support are therefore essential and should not be overlooked.

### Fetal outcome

The overall fetal death rate in our analysis was 34.1% and was did not differ between trimesters; this is in agreement with the study of van Steenbergen *et al.* [8]. The key question is how these high rates can be reduced. Diagnosing valve thrombosis or endocarditis earlier might improve the outcome allowing time for non-surgical options such as thrombolysis and antibiotic treatment to be effective. Alerting not only physicians, but also pregnant patients, especially those with a mechanical valve *in situ*, to the early symptoms and signs of infection or valve thrombosis would promote earlier presentation and diagnosis. We found differences in the incidence of fetal monitoring during valve replacement among trimesters, as expected due to the viability of the fetus. The current ESC pregnancy guidelines recommend monitoring of the fetal heart rate during CPB [4]. Monitoring during the earlier stages of pregnancy might identify when uterine perfusion is compromised and prompt alterations in the anaesthetic of perfusion parameters. We included all cases from the literature, but the numbers were still limited, meaning that although we found marked differences in fetal death rates between those pregnancies that were monitored during surgery (21% vs 38% without fetal monitoring), this was not significant. However, we believe that external monitoring of the fetal heart rate should be considered. Fetal bradycardias resulting from uteroplacental hypoperfusion due to medications, maternal malpositioning or the CPB itself can be detected and corrected [12]. Interventions include intravenous fluid administration, ephedrine and/or changing the maternal position to left lateral tilt. In case of a viable fetus, if there is no normalization of the fetal heart rate, an emergency Cesarean section should be considered. A side note here is that external fetal heart rate monitoring is sometimes challenging at early gestational age with an increased possibility of signal loss and poor quality of the monitoring [13]. Fetal mortality risk increases with the presence of uterine contractions; therefore, monitoring of uterine activity is also recommended and tocolytic treatment can be instituted if indicated. We found no differences in the occurrence of fetal death and details of CPB (i.e. CPB duration, cross-clamp time, hypothermia, pulsatile flow, pump flow and mean arterial pressure); however, the CPB details in the included cases were rarely reported and insufficient to draw conclusions. Previous studies showed more favourable fetal outcomes with the use of pulsatile and high flow CPB (>2.5 l/min/m^2^), a mean arterial pressure >70–75 mmHg and avoidance of hypothermia [4, 5, 12, 14–16]. Performing a Cesarean section prior to valve replacement should be considered in the presence of an independently viable fetus, as previous research shows that Cesarean section prior to cardiac surgery was associated with a lower fetal mortality [8]. Further, our data show that the fetal death rate was still 27% when the valve replacement was performed during the 3rd trimester. With improved fetal outcomes after early delivery, the balance should shift towards Cesarean section in all cases with need for cardiac surgery beyond viable gestation age, while the long-term consequences of preterm birth should not be overlooked [17]. This emphasizes the importance of collaboration between different disciplines, including at least the cardiologist, cardiothoracic surgeon, anesthesiologist, obstetrician and neonatologist to discuss both maternal and fetal risks and survival rates.

### Changes over time and areas for improvement

We compared the surgery characteristics and outcomes between the cases published before and after 2010 and found no statistical differences in maternal and fetal death rates. Since there are only limited differences in the surgical characteristics between the cases published before 2010 and after 2010, this seems to indicate that there has been at least no improvement in survival after valve replacement during pregnancy over time. This shows that it is even more important to focus on the cause of valve dysfunction to prevent the need for valve replacement during pregnancy. In case of valve dysfunction, performing 1st valve repair or replacement before becoming pregnant should be considered; however, the indication in a significant portion of the cases in current study was acute valve dysfunction and valve thrombosis. This emphasizes the importance of preconception assessment and planning to agree a plan for anticoagulation and optimization of woman’s clinical state. Given the high risk of maternal and fetal mortality and complications, the indication for valve replacement is very important. If the situation is life-threatening and no other treatment options are available, valve replacement can be the only option. Otherwise, alternatives such as thrombolysis (in case of valve thrombosis), performing a cesarean section prior to valve replacement in the case of a viable fetus, or a percutaneous intervention should be considered. Several case reports have been published on transcatheter aortic valve replacement in pregnant women, showing promising results, and may offer a solution in some cases [18]. In addition, complications often occurred with an existing mechanical valve, and when a native valve was replaced, a mechanical valve was typically chosen. However, pregnancy outcomes are significantly better in women with a tissue valve compared to those with a mechanical valve [19]. Therefore, there is a compelling case for choosing a tissue valve over a mechanical valve in women of reproductive age, despite the limited lifespan, in order to avoid complications associated with anticoagulation and the risk of valve thrombosis during pregnancy.

### Anticoagulation

No anticoagulation regimen during pregnancy in women with a prosthetic valve has been shown to be clearly superior [6, 20]. Due to the potential teratogenicity of vitamin K antagonists (VKAs), low-molecular-weight heparin (LMWH) or unfractionated heparin (UFH) is often used during the 1st trimester. A disadvantage of both forms of heparin is that they are associated with a higher chance of thromboembolic complications [20]. Due to limited data on anticoagulation in the other cases in our review, no clear conclusion can be made on anticoagulation and risks; however, it is clear that a randomized controlled trial is required to establish which regimen is superior not only in terms of maternal outcomes but also in terms of fetal outcomes.

### LMIC versus HIC

We compared the surgery characteristics and outcomes between LMICs and HICs and found differences in type and position of the valve and indication for valve replacement. We believe these differences are related to the aetiology of the valvular heart disease, as 42–85% of all pregnancies in women with heart disease involved rheumatic heart disease which primarily affects the mitral valve, followed by the aortic valve [21–23]. Rheumatic heart disease is more common in LMICs, while congenital heart disease is more frequently diagnosed and treated in HICs. Although not statistically significant, maternal death seems more common after valve replacement during pregnancy in LMICs (15% vs 3% in HICs). This is also found by previous registries on pregnancy outcomes in women with heart disease [3, 21, 24]. This may be related to reduced accessibility of care, resulting in a later presentation and hence more advanced disease. However, the underlying cause of the heart disease might also play a role. Further, fetal monitoring was less frequently performed in LMICs, perhaps reflecting the scarcity of resources to monitor the fetus during surgery in these countries. Also, the timing of surgery could play a role, with more valve replacements during the 3rd trimester in cases from HICs than from LMICs (18% vs 9%), and therefore a higher probability that the fetus will be monitored than in the 1st and early 2nd trimester. Despite this difference in fetal monitoring, we found no difference in fetal death rates between LMICs and HICs.

### Limitations

Our study has several limitations. First, there was a significant amount of missing data, particularly on details of CPB and anticoagulation as stated above. However, we had a low number of missing data on the other variables. Second, as with other reviews based on case reports and case series, selection and publication bias are likely with a strong possibility that cases with a less favourable outcome (i.e. maternal and/or fetal death) are not published, which results in an underestimation of the risk of maternal and fetal death after valve replacement with a fetus *in utero*. It follows from this that overall the outcomes may be even worse.

## CONCLUSIONS

Valve replacement during pregnancy with the fetus *in utero* is associated with a high maternal and extremely high fetal mortality, with the highest risks in replacement of an existing mitral prosthetic valve. These results emphasis the great importance of prevention of prosthetic valve complications and the earlier diagnosis of valve complications allowing for earlier intervention and hopefully the use of non-surgical approaches.

## Supplementary Material

ezaf180_Supplementary_Data

## Data Availability

The de-identified participant data are available from the corresponding author on reasonable request.

## ABBREVIATIONS

CPB: Cardiopulmonary bypass
ESC: European Society of Cardiology
HIC: High-income country
LMIC: Low-or-middle-income country
LMWH: Low-molecular-weight heparin
ROPAC: Registry Of Pregnancy And Cardiac disease
UFH: Unfractionated heparin
VKA: Vitamin K antagonist
