# Supplementary material for: Valve replacement during pregnancy: literature review including new data from the Registry Of Pregnancy And Cardiac disease III
Source: Eur J Cardiothorac Surg. 2025 Jun 20;67(7):ezaf180. doi: 10.1093/ejcts/ezaf180 (PMC12245401; doi:10.1093/ejcts/ezaf180)
Supplement: ezaf180_Supplementary_Data [file ezaf180_supplementary_data.docx]

**Supplemental material**

**ROPAC Investigators**

***EORP Oversight Committee***

**2014-2016**: R. Ferrari, IT (Chair); A. Alonso, ES; J. Bax, NL; C. Blomström-Lundqvist, SE; S. Gielen, DE; P. Lancellotti, BE; A.P. Maggioni, IT; N. Maniadakis, GR; F. Pinto, PT; F. Ruschitzka, CH; L. Tavazzi, IT; P. Vardas, GR; F. Weidinger, AT; U. Zeymer, DE. **2016-2018**: A. Vahanian, FR (Chair); A. Budaj, PL; N. Dagres, DE; N. Danchin, FR; V. Delgado, NL; J. Emberson, GB; O. Friberg, SE; C.P. Gale, GB; G. Heyndrickx, BE; B. Iung, FR; S. James, SE; A.P. Kappetein, NL; A.P. Maggioni, IT; N. Maniadakis, GR; K.V. Nagy, HU; G. Parati, IT; A-S. Petronio, IT; M. Pietila, FI; E. Prescott, DK; F. Ruschitzka, CH; F. Van de Werf, BE; F. Weidinger, AT; U. Zeymer, DE. **2018-2020**: C.P. Gale, GB (Chair); B. Beleslin, RS; A. Budaj, PL; O. Chioncel, RO; N. Dagres, DE; N. Danchin, FR; J. Emberson, GB; D. Erlinge, SE; M. Glikson, IL; A. Gray, GB; M. Kayikcioglu, TR; A.P. Maggioni, IT; K.V. Nagy, HU; A. Nedoshivin, RU; A-P. Petronio, IT; J.W. Roos-Hesselink, NL; L. Wallentin, SE; U. Zeymer, DE. **2020-2022**: B.A. Popescu, RO (Chair); D. Adlam, GB; A.L.P. Caforio, IT; D. Capodanno, IT; M. Dweck, GB; D. Erlinge, SE; M. Glikson, IL; J. Hausleiter, DE; B. Iung, FR; M. Kayikcioglu, TR; P. Ludman, GB; L. Lund, SE; A.P. Maggioni, IT; S. Matskeplishvili, RU; B. Meder, DE; K.V. Nagy, HU; A. Nedoshivin, RU; D. Neglia, IT; A.A. Pasquet, BE; J.W. Roos-Hesselink, NL; F.J. Rossello, ES; S.M. Shaheen, EG; A. Torbica, IT.

***Executive Committee***

Jolien Roos-Hesselink, The Netherlands (Co-chair); Roger Hall, United Kingdom (Co-chair); William Parsonage, Australia; Werner Budts, Belgium; Julie de Backer, Belgium; Jasmin Grewal, Canada; Ariane Marelli, Canada; Guillaume Jondeau, France; Mark Johnson, United Kingdom; Catherine Otto, United States; Karen Sliwa, South Africa; Aldo Maggioni, Italy.

***Investigators***
**Armenia:***Yerevan:*K. Vardanyan, A. Melkonyan, H. Lachikyan, K. Hakobyan, M. Mazmanian, *Yerevan:*H. Hayrapetyan, A. Tavaracyan, H. Poghosyan, R. Hovhannisyan, S. Sahakyan, S. Martirosyan, **Australia:***St Leonards:*J. Harris, **Belgium:***Brussels:*A. Pasquet, *Brussels:*M. Morissens, T. Besse-Hammer, B. Dumoulin, *Gent:*J. De Backer, L. Campens, L. Demulier, M. De Hosson, *Leuven:*W. Budts, A. Van de Bruaene, A. Rampelberg, E. Troost, L. Roggen, P. De Meester, **Botswana:***Gaborone:*J.C. Mwita, E. Tefera, L. Kontle, **Canada:***Montreal:*A. Marelli, I. Malhamé, *Vancouver:*J. Grewal, M. Janzen, **Cuba:***La Habana:*P.A. Román Rubio, R. Vasallo Peraza, G. Vázquez Hernández, J.E. Pérez Torga, Y. Gil Jiménez, M. Meluzá Martín, **Egypt:***Cairo:*R. Almaleh, *Cairo:*G. Youssef, K. Sorour, **Ethiopia:***Addis Ababa:*S. Abebe, D. Mekonnen, C. Fekadu, D. Yadeta, **France:***Bron:*S. Dupuis-Girod, L. Delagrange, *Lille:*M. Richardson, L. Ghesquiere, O. Domanski, M. Gonzalez Estevez, Y. Ould Hamoud, S. Gautier, L. Marsili, *Marseille:*L. Bal-Theoleyre, S. Palazzolo, *Paris:*M. Ladouceur, *Paris:*G. Jondeau, A. Bourgeois Moine, L. Eliaou, O. Milleron, M. Tchitchinadze, *Toulouse:*Y. Dulac, C. Karsenty, N. Souletie, F. Bajanca, **Germany:***Hamburg:*C. Rickers, S. Blankenberg, C. Sinning, C. Magnussen, E. Zengin, G. Mueller, R. Schnabel, Y. Von Kodolitsch, R. Kozlik-Feldmann, *Muenster:*H. Baumgartner, R. Schmidt, A. Hellige, A. Rietkötter, **Greece:***Athens:*M. Spartalis, *Athens:*A.A. Frogoudaki, *Thessaloniki:*A. Arvanitaki, A. Baroutidou, G. Giannakoulas, C. Karvounis, **India:***Chennai:*J.P. Gnanaraj, A.P. Steaphen, T. Ethirajan, K. Kannan, V. Subramanian, A. Surendran, J. Gnanasekaran, V. Natarajalingam, S. Balasubramani, **Iraq:***Bagdad:*H. Ali Farhan, I.F. Yaseen, **Italy:***Bologna:*E. Mariucci, C. Ciuca, *Massa:*F. Marchi, G. Benedetti, M. Baroni, P. Festa, A. Parlanti, *Naples:*G. Scognamiglio, F. Fusco, B. Sarubbi, *Trieste:*M. Merlo, B. D'Agata Mottolese, C. Carriere, G. Sinagra, M. Bobbo, F. Ramani, *Turin:*F.M. Comoglio, R. Bordese, A. Pagano, N. Montali, V. Donvito, C.A. Remolif, F. Petey, **Netherlands:***Amsterdam:*B. Bouma, S. Chamuleau, D. Robbers-Visser, *Amsterdam:*T. Konings, H. Dronkert, *Breda:*D. Segers, *Nijmegen:*R. Van Kimmenade, *Utrecht:*H. Van Der Zwaan, G. Tjalling Sieswerda, A. Evers, T. Schaap, **Pakistan:***Karachi:*K. Bano, H. Yasmeen, K. Amir, N. Patel, P. Akhter, R. Khan, A. Shakeel, S. Mahar, S. Habib, **Poland:***Lodz:*M. Lelonek, *Warsaw:*P. Hoffman, M. Lipczynska, **Portugal:***Lisbon:*L. De Sousa, V. Ferreira, T. Mano, M. Selas, R. Cruz Ferreira, **Russian Federation:***Saint Petersburg:*E. Shlyakhto, O. Irtyuga, G. Sefieva, K. Malikov, T. Pervunina, U. Shadrina, **Saudi Arabia:***Jeddah:*A. Kinsara, *Riyadh:*D. Galzerano, H. Al Sergani, W. Kurdi, N. Kholaif, O. Vriz, A. Alhamshari, A. Alsaigh, O. Ahmad, S. Alzaher, B. Alamro, **South Africa:***Cape Town:*K. Sliwa, F. Jakoet-Bassier, **Spain:***Barcelona:*L. Galian-Gay, A. Pijuan-Domenech, B. Miranda-Barrio, B. Gordon, **Sweden:***Goteborg:*E. Furenäs, *Lund:*J. Hlebowicz, F. Wedlund, *Stockholm:*E. Nagy, E. Mattsson, M. Majczuk Sennstrom, P. Sörensson, *Uppsala:*C. Christersson, A. Lutvica, B. Jönelid, T. Achter, K. Junus, H. Gärdesten Wall, M. Andreasson, **Switzerland:***Basel:*D. Tobler, *Genève:*J. Bouchardy, F. Brand, C. Blanche, *Lausanne:*J. Bouchardy, T. Rutz, F. Brand, **Türkiye:***Ankara:*U. Canpolat, Y.Z. Sener, N. Ozer, *Istanbul:*E. Ayduk Gövdeli, Z. Bugra, B. Umman, P. Karaca Özer, D. Baykiz, *Istanbul:*D. Mutlu, H. Yalman, B. Kilickiran Avci, *Istanbul/turkey:*S. Catirli Enar, O. Batukan Esen, D. Oksen, **Ukraine:***Kyiv:*V. Lazoryshynets, S. Siromakha, Y. Davydova, A. Limanska, I. Zinovchyk, V. Kravchenko, B. Kravchuk, O. Kravets, N. Volkova, O. Mazur, O. Beregovyi, **United Arab Emirates:***Abu Dhabi:*B.T. Salih, W.A.R. Al Mahmeed, S. Wani, F.S. Mohamed Farook, G. Al Mansoori, **United States:***Houston:*S. Prakash, R. Afifi, D. Milewicz, A. Cecchi, *Lexington, Kentucky:*G. Wells, D. Sparks, *Minneapolis, Mn:*W. Wagner, C. Bigelow, L. Colicchia, T. Jentink, M. Loichinger, R. Saxena, W. Wunderlich, C. Longtin, P. Klopper, R. Gobar, *New Haven:*J. Chou, K. Campbell, R. Elder, *New York:*D. Halpern, A. Hausvater, H. Reynolds, N. Bhalla, A. Small, J. Feinberg, P. Panday, *Phoenix:*J. Awerbach, J. Porche, S. Stack, *Portland:*L. Mcgrath, A. Khan, E. Pare, P. Woods, C. Broberg, K. Gibbins, K. Brookfield, *Stony Brook:*M. Al-Sadawi, A. Cove, N. Mann.

**References included case series and case reports**

**(1-48)**

Bahary CM, Ninio A, Gorodesky IG, Neri A. Tococardiography in pregnancy during extracorporeal bypass for mitral valve replacement. Isr J Med Sci. 1980;16(5):395-7.

Botta L, Merati R, Vignati G, Orcese CA, De Chiara B, Cannata A, et al. Mitral valve endocarditis due to Abiotrophia defectiva in a 14th week pregnant woman. Interact Cardiovasc Thorac Surg. 2016;22(1):112-4.

Boulemden A, Malin GL, Wallace SVF, Mahmoud A, Smith WHT, Szafranek AA. Mechanical Mitral Valve Replacement during the 2nd Trimester of Pregnancy. Tex Heart Inst J. 2018;45(1):31-4.

Carnero-Alcazar M, Reguillo-Lacruz F, Montes-Villalobos L, Rodriguez-Hernandez JE. Mechanical prosthetic mitral valve thrombosis in a first trimester pregnant woman. Interact Cardiovasc Thorac Surg. 2010;10(1):116-8.

Cunha CR, Santos PC, Castineira CP, Pereira FS. Heart valve replacement during pregnancy. Rev Bras Cir Cardiovasc. 2007;22(4):498-500.

Deviri E, Yechezkel M, Levinsky L, Vidne BA, Levy MJ. Calcification of a porcine valve xenograft during pregnancy--a case report and review of the literature. Thorac Cardiovasc Surg. 1984;32(4):266-8.

Dubrey SW, Kohli SK, Grocott-Mason R, Dhanjal MK, Punjabi PP, Nihoyannopoulos P, Nelson-Piercy C. Rheumatic mitral valve disease in pregnancy. Br J Hosp Med (Lond). 2010;71(5):294.

Eilen B, Kaiser IH, Becker RM, Cohen MN. Aortic valve replacement in the third trimester of pregnancy: case report and review of the literature. Obstet Gynecol. 1981;57(1):119-21.

Elassy SM, Elmidany AA, Elbawab HY. Urgent cardiac surgery during pregnancy: a continuous challenge. Ann Thorac Surg. 2014;97(5):1624-9.

Elsayed AAA, Abdelaal KM, Abdelghaffar AMM, Mohamed EEH, Mahran TMA, Ahmed MSM, et al. Poor Outcome of Surgical Management of Acute Malfunctioning Mechanical Mitral Valve During Pregnancy. Should Centers with Limited Resources Find Different Options? Heart Surg Forum. 2019;22(5):E405-E10.

Foolchand S, Ramnarain H. Prosthetic heart valve thrombosis in pregnancy: a case series on acute management. Cardiovasc J Afr. 2021;32(4):228-32.

Golden LP. Aortic valve repair and arch replacement during pregnancy: a case report. AANA J. 1996;64(3):243-54.

Gupta R, Ranchal P, Harburger J. Mechanical Valve Thrombosis in a Pregnant Patient: A Case of Therapeutic Failure. Cureus. 2019;11(9):e5615.

Iscan ZH, Mavioglu L, Vural KM, Kucuker S, Birincioglu L. Cardiac surgery during pregnancy. J Heart Valve Dis. 2006;15(5):686-90.

Izquierdo LA, Kushnir O, Knieriem K, Wernley JA, Curet LB. Effect of mitral valve prosthetic surgery on the outcome of a growth-retarded fetus. A case report. Am J Obstet Gynecol. 1990;163(2):584-6.

Jadhav KP, Sridhar KS, Kandi S, Jariwala PV. A 'dreaded' complication of stuck prosthetic valve in the first trimester pregnancy. J Cardiol Cases. 2023;28(6):261-4.

Jafferani A, Malik A, Khawaja RD, Sheikh L, Sharif H. Surgical management of valvular heart diseases in pregnancy. Eur J Obstet Gynecol Reprod Biol. 2011;159(1):91-4.

James C, Felix C. A 30-year-old pregnant woman with pulmonary edema from a clotted mechanical aortic valve. J Emerg Nurs. 1998;24(2):123-6.

Johnston RC, Swank ML, Shrivastava VK, Hameed AB. Patient-prosthesis mismatch in pregnancy. Obstet Gynecol. 2014;123(2 Pt 2 Suppl 2):441-3.

Kaoutzanis C, Evangelakis E, Kokkinos C, Kaoutzanis G. Urgent aortic valve replacement for infective endocarditis during the 23rd week of pregnancy. Gen Thorac Cardiovasc Surg. 2013;61(5):296-300.

Khan N, Pumphrey C, Clarke J, Jahangiri M. Aortic root replacement in pregnancy. J R Soc Med. 2003;96(11):551-2.

Kikon M, Dutta Choudhury K, Prakash N, Gupta A, Grover V, Kumar Gupta V. Mitral valve replacement in a young pregnant woman: a case report and review of literature. Res Cardiovasc Med. 2014;3(2):e17561.

Kisat M, Fatimi SH, Sheikh L, Samad K. Mitral valve replacement in a twin pregnancy. J Obstet Gynaecol Res. 2011;37(7):916-8.

Kole SD, Jain SM, Walia A, Sharma M. Cardiopulmonary bypass in pregnancy. Ann Thorac Surg. 1997;63(3):915-6.

Korsten HH, Van Zundert AA, Mooij PN, De Jong PA, Bavinck JH. Emergency aortic valve replacement in the 24th-week of pregnancy. Acta Anaesthesiol Belg. 1989;40(3):201-5.

Lin TY, Chiu KM, Shieh JS, Chu SH. Emergency redo mitral valve replacement in a pregnant woman at third trimester: case report and literature review. Circ J. 2008;72(10):1715-7.

Marcoux J, Rosin M, Mycyk T. CPB-assisted aortic valve replacement in a pregnant 27-year-old with endocarditis. Perfusion. 2009;24(5):361-4.

Masuda Z, Miyamoto Y, Une D, Inoue Y, Tateishi A, Yokota Y, et al. Acute mitral valve endocarditis at the 24th gestational week. Gen Thorac Cardiovasc Surg. 2020;68(12):1457-60.

Miller M, Buchanan N, Cane RD, Kinsley R. Two mitral valve replacements during the course of a single pregnancy. Intensive Care Med. 1978;4(1):41-2.

Mokgwathi GTL, E.M.; Ogunbanjo, G.A. Positive maternal and foetal outcomes after cardiopulmonary bypass surgery in a parturient with severe mitral valve disease. Southern African Journal of Anaesthesia and Analgesia. 2011;17(4).

Mooij PN, de Jong PA, Bavinck JH, Korsten HH, Bonnier JJ, Berendes JN. Aortic valve replacement in the second trimester of pregnancy: a case report. Eur J Obstet Gynecol Reprod Biol. 1988;29(4):347-52.

Mora CT, Grunewald KE. Reoperative aortic and mitral prosthetic valve replacement in the third trimester of pregnancy. J Cardiothorac Anesth. 1987;1(4):313-7.

Motherwell DW, Ramsay JE, Craig SR, Brady AJ. Thrombosed prosthetic valve in a 32-week pregnant 20-year-old woman. Can J Cardiol. 2007;23(6):444.

Munoz-Mendoza J, Pinto Miranda V, Tanawuttiwat T, Badiye A, Chaparro SV. Severe bioprosthetic mitral valve stenosis in pregnancy. Gen Thorac Cardiovasc Surg. 2016;64(1):38-42.

Muretti M, Torre TM, Mauri R, Trunfio R, Moschovitis G, Siclari F. Mitral valve replacement in pregnancy: a successful strategy for fetal survival. J Heart Valve Dis. 2010;19(6):789-91.

O'Donnell D, Gillmer DJ, Mitha AS. Aortic and mitral valve replacement for bacterial endocarditis in pregnancy. A case report. S Afr Med J. 1983;64(27):1074.

Paulus DA, Layon AJ, Mayfield WR, D'Amico R, Taylor WJ, James CF. Intrauterine pregnancy and aortic valve replacement. J Clin Anesth. 1995;7(4):338-46.

Pitfield AF, Bedard A, Bashir J, Bruce S, Augoustides JG, Cormican DS, et al. Anesthetic Management for Cardiac Surgery During Pregnancy Complicated by Postoperative Threatened Abortion. J Cardiothorac Vasc Anesth. 2023;37(1):158-66.

Price JW, Klas M. Emergency tricuspid valve replacement during pregnancy. J Clin Anesth. 2010;22(6):454-9.

Sathananthan G, Johal N, Grewal J. A case report: mechanical tricuspid valve thrombosis necessitating cardiac surgery during pregnancy. Eur Heart J Case Rep. 2019;3(2).

Siddiqui KM, Khan FH. Anaesthetic management of a pregnant patient for aortic valve replacement. J Pak Med Assoc. 2008;58(9):521-2.

Sutton SW, Duncan MA, Chase VA, Marce RJ, Meyers TP, Wood RE. Cardiopulmonary bypass and mitral valve replacement during pregnancy. Perfusion. 2005;20(6):359-68.

Tehrani H, Masroor S, Lombardi P, Rosenkranz E, Salerno T. Beating heart aortic valve replacement in a pregnant patient. J Card Surg. 2004;19(1):57-8.

Tripp HF, Stiegel RM, Coyle JP. The use of pulsatile perfusion during aortic valve replacement in pregnancy. Ann Thorac Surg. 1999;67(4):1169-71.

van Steenbergen GJ, Tsang QHY, van der Heijden OWH, van Kimmenade RRJ, Bouwmeester S, Li WWL, Verhagen A. Emergency Aortic Valve Replacement in a 12-Week Pregnant Patient. JACC Case Rep. 2020;2(1):107-11.

Vosa C, Renzulli A, Festa M, Sante P, Micheletti E. Cardiac valve replacement during pregnancy. Report of two cases. Ital J Surg Sci. 1988;18(2):175-7.

Vrkocova KP, J.; Veiser, T.; Homza, M. Rare case of a patient with recurrent thrombosis of a mechanical valve during pregnancy. Cor et Vasa. 2016;58:e451-e6.

Zellner JL, Kribbs SB, Dorman BH, Spinale FG. Cardiopulmonary bypass in a gravid patient: perioperative changes in endothelin levels. Ann Thorac Surg. 1998;66(1):268-70.

**Supplementary Table 1**

**Search strategy**

Medline

(exp * Heart Valve Prosthesis Implantation / OR exp * Heart Valve Prosthesis / OR (((heart-valve* OR cardiac-valve* OR aortic-valve* OR valve* OR valvular* OR mitral* OR tricuspid) ADJ6 (replacement* OR implant* OR prosthe* OR bioprosthe* OR mechanical* OR artificial* OR percutaneous*)) OR Ross-procedure OR tavi OR tavr).ti.) AND (exp Pregnancy / OR Pregnant Women / OR * Pregnancy Outcome / OR exp Pregnancy Complications / OR * Delivery, Obstetric / OR * Parturition / OR (pregnan* OR (obstetr* ADJ3 (deliver*)) OR labor OR labour* OR childbirth* OR child-birth* OR cesarean OR caesarean OR c-section* OR ((fetal* OR foetal* OR fetus* OR foetus* OR maternal* OR mother*) ADJ3 outcome*) OR abortion* OR miscarriage* OR live-birth* OR still-birth* OR livebirth* OR stillbirth*).ab,ti,kw.) NOT (exp animals/ NOT humans/) AND english.la.

Embase

('heart valve replacement'/mj/exp OR 'heart valve prosthesis'/mj/exp OR (((heart-valve* OR cardiac-valve* OR aortic-valve* OR valve* OR valvular* OR mitral* OR tricuspid) NEAR/6 (replacement* OR implant* OR prosthe* OR bioprosthe* OR mechanical* OR artificial* OR percutaneous*)) OR Ross-procedure OR tavi OR tavr):ti) AND (pregnancy/exp OR 'pregnant woman'/de OR 'pregnancy outcome'/mj/de OR 'pregnancy complication'/mj/exp OR 'pregnancy disorder'/mj/exp OR 'obstetric delivery'/mj/exp OR childbirth/mj/de OR (pregnan* OR (obstetr* NEAR/3 (deliver*)) OR labor OR labour* OR childbirth* OR child-birth* OR cesarean OR caesarean OR c-section* OR ((fetal* OR foetal* OR fetus* OR foetus* OR maternal* OR mother*) NEAR/3 outcome*) OR abortion* OR miscarriage* OR live-birth* OR still-birth* OR livebirth* OR stillbirth*):Ab,ti,kw) NOT [conference abstract]/lim NOT ([animals]/lim NOT [humans]/lim) AND [english]/lim

Cochrane

((((heart-valve* OR cardiac-valve* OR aortic-valve* OR valve* OR valvular* OR mitral* OR tricuspid) NEAR/6 (replacement* OR implant* OR prosthe* OR bioprosthe* OR mechanical* OR artificial* OR percutaneous*)) OR Ross-procedure OR tavi OR tavr):ti) AND ((pregnan* OR (obstetr* NEAR/3 (deliver*)) OR labor OR labour* OR childbirth* OR child-birth* OR cesarean OR caesarean OR c-section* OR ((fetal* OR foetal* OR fetus* OR foetus* OR maternal* OR mother*) NEAR/3 outcome*) OR abortion* OR miscarriage* OR live-birth* OR still-birth* OR livebirth* OR stillbirth*):Ab,ti,kw)

**Screening Methods and Data collection**

Titles and abstracts of the records were screened independently by two reviewers (J.A.v.d.Z, P.P), followed by full-text screening of the articles that were eligible. In case of disagreement, consensus was reached through discussion and if necessary, a third reviewer was consulted (J.W.R-H). Studies were included if they included patients who underwent surgical valve replacement during pregnancy. In addition, studies had to be written in English. Studies were excluded if they included cases from previous published studies (e.g. reviews), transcatheter procedures, cases in which fetal death was observed prior to valve replacement, and cases in which a Cesarean section was performed prior to valve replacement. Two reviewers independently collected data from the eligible studies and disagreements were resolved through discussion with a third reviewer (J.A.v.d.Z, P.P, J.W.R-H). Variables collected were study characteristics (first author, year of publication), gestational age at valve replacement, surgery details (type of surgery, cardiac bypass time, aortic clamp time, pump flow, mean arterial pressure, temperature, fetal monitoring during surgery), maternal outcomes, fetal outcomes, and delivery details (gestational age at delivery and mode of delivery).

|  | Supplementary Table 2. Procedure, details of ROPAC III cases | | | | | | | | |
| --- | --- | --- | --- | --- | --- | --- | --- | --- | --- |
|  | Bypass time (min) | Aortic cross-clamping (min) | Intraoperative blood loss (ml) | Temperature (^o^C) | RBC transfusion (ml) | Plasma transfusion (ml) | Platelet transfusion (ml) |  |  |
|  | FIRST VALVE DURING PREGNANCY | | | | | | | |  |
| 1. | - | - | - | - | - | - | - |  |  |
| 2. | 124 | 92 | 200 | 34 | 630 | 400 | 40 |  |  |
| 3. | 128 | 104 | 300 | 34 | 680 | 400 | 150 |  |  |
| 4. | 131 | 109 | 200 | 35 | 1143 | 600 | - |  |  |
| 5. | - | - | - | - | - | - | - |  |  |
| 6. | 107 | 88 | 200 | 35 | 333 | 250 | - |  |  |
| 7. | - | - | - | - | - | - | - |  |  |
|  | REPLACEMENT OF PROSTHETIC VALVE DURING PREGNANCY | | | | | | | |  |
| 8. | - | - | - | - | - | - | - |  |  |
| 9. | 67 | 54 | - | 35 | 200 | - | - |  |  |
| 10. | - | - | - | - | - | - | - |  |  |
| 11. | - | - | - | - | - | - | - |  |  |

| Supplementary Table 3. Anticoagulation during pregnancy with target levels, details of ROPAC III cases | | | | | | |
| --- | --- | --- | --- | --- | --- | --- |
|  | **Before pregnancy** | **0-6 weeks** | **6-14 weeks** | **14-36 weeks** | **36 weeks - delivery** | **Postpartum** |
| FIRST PROSTHETIC VALVE DURING PREGNANCY | | | | | | |
| 1. | None | Warfarin (3mg/day) INR 2.5-3.5 | Warfarin (3mg/day) INR 2.5-3.5 | Warfarin (3mg/day) INR 2.5-3.5 | UFH | LMWH, switch to Warfarin (timing of switch unknown) |
| 2. | None | None | None | Warfarin (6mg/day)  INR 2.0-3.0 | LMWH (enoxaparin)  Anti-Xa 0.4-0.7 | LMWH (enoxaparin) Anti-Xa 0.4-0.7 for 14 days, then Warfarin (6mg/day) INR 2.0-3.0 |
| 3. | None | None | None | Warfarin (5mg/day)  INR 2.0-3.0 | LMWH (enoxaparin)  Anti-Xa 0.4-0.7 | LMWH (enoxaparin) Anti-Xa 0.4-0.7 for 14 days, then Warfarin (5mg/day) INR 2.0-3.0 |
| 4. | None | None | None | Warfarin (6mg/day)  INR 2.0-3.0 | LMWH (enoxaparin)  Anti-Xa 0.4-0.7 | LMWH (enoxaparin) Anti-Xa 0.4-0.7 for 14 days, then Warfarin (6mg/day) INR 2.0-3.0 |
| 5. | None | None | None | Warfarin (6mg/day)  INR 2.5-3.5 | LMWH (enoxaparin)  Anti-Xa 0.4-0.7 | LMWH (enoxaparin) Anti-Xa 0.4-0.7 for 14 days, then Warfarin (7.5mg/day) INR 2.0-3.0 |
| 6. | None | None | None | Warfarin (3mg/day)  INR 2.0-3.0 | LMWH (enoxaparin)  Anti-Xa 0.4-0.7 | LMWH (enoxaparin) Anti-Xa 0.4-0.7 for 14 days, then Warfarin (3mg/day) INR 2.0-3.0 |
| 7. | None | None | None | Warfarin (5mg/day) INR | LMWH + Aspirin (150mg/day) | LMWH, switch to Warfarin (5mg/day) (timing of switch unknown) |
| REPLACEMENT OF PROSTHETIC VALVE DURING PREGNANCY | | | | | | |
| 8. | Warfarin (8mg/day) INR 2.5-3.5 | Switch to UFH early in pregnancy | Patient died | - | - | - |
| 9. | N/A | LWMH | LMWH, UFH at time of valve thrombosis, PTT ratio 2.0-2.5 + Aspirin | Acenocoumarol (4mg/day) INR 3.5-4.0 | UFH | N/A |
| 10. | Warfarin (13.5mg/day) | LMWH (enoxaparin) Anti-Xa>0.9, biweekly measurements | LMWH (enoxaparin) Anti-Xa>0.9, biweekly measurements | 14-15 weeks: LMWH (enoxaparin) Anti-Xa>0.9, biweekly measurements. 15-18 weeks: UFH + aspirin 75mg/day. 18 weeks till surgery: LMWH (enoxaparin) Anti-Xa 1.0-1.2 + Aspirin 75mg/day. Surgery-36 weeks: Warfarin (8mg/day) INR 2.5-3.5 + Aspirin 75mg/day | LMWH (enoxaparin) | LMWH (enoxaparin) for 15 days, then Warfarin (13mg/day) (timing of switch unknown) |
| 11. | Warfarin (18mg/day) | Switch to LMWH early in pregnancy  Anti-Xa 0.25-0.50 | LMWH  Anti-Xa 0.6-1.0  After surgery: warfarin | Warfarin | Preterm delivery (27 weeks) | LMWH, switch to Warfarin (timing of switch unknown) |

**Supplementary Table 4.** Cases of valve replacement during pregnancy published in literature

| Case no. | First author (year) | Maternal age, years | Indication for valve replacement | GA at surgery, weeks | Surgery | CBP time (clamp time), min | Pump flow, L/min/m^2^ (P, NP) | MAP, mmHg | T, °C | Fetal monitoring during surgery | Maternal outcome | Fetal outcome | GA at delivery, weeks | Delivery |
| --- | --- | --- | --- | --- | --- | --- | --- | --- | --- | --- | --- | --- | --- | --- |
| 1. | Bahary  (1980) | 23 | RHD, MS | 32 | MVR (?) | 37 | - | 70 | - | Yes, decelerations | Good | Good | 40 | CS |
| 2. | Botta  (2015) | 39 | IE, MR | 18 | MVR (m) | 76 (40) | 130% of theoretical value | - | N | Yes, good | Good | Good | 38 | CS |
| 3. | Boulemden  (2018) | 44 | MS | 23 | MVR (m) | - | - | 70 | 36-37 | No | Good | Good | 38 | VD |
| 4. | Carnero  (2009) | 31 | PVT (tPA-) | 9 | MVRR (m) | - | - | 70 | >34 | No | Good | Good | - | - |
| 5. | Da Cunha  (2007) | 21 | MS, AR | 20 | AVR (t) + MVRR (t) | 105 | - | - | 35 | No | Good | Good | 31 | CS |
| 6.  † | Deviri  (1984) | 20 | MS | 32 | MVRR (m) | - | - | - | - | No | Death (PP 12m)  Massive brain embolization | Good | 37 | VD |
| 7. | Dubrey  (2010) | 32 | RHD, MS | 21 | MVR (m) | - | - | - | - | No | Pre-eclampsia or HELLP syndrome  Good | Death (POD2) | N/A | N/A |
| 8. | Eilen  (1981) | 25 | RHD, AR | 31 | AVR (t) | - | - | - | - | Yes, bradycardias | Good | Good | 40 | VD |
| 9. | Elassy  (2014) | 27 | RHD, AS | 14 | AVR (m) | 53 (27) | >2.4 (P) | >70 | - | No | Good | Death (after) | N/A | N/A |
| 10. | Elassy  (2014) | 31 | RHD, MS | 26 | MVR (m) | 65 (38) | >2.4 (P) | >70 | - | No | Good | Good | Term | VD |
| 11. | Elassy  (2014) | 30 | RHD, MS | 27 | MVR (m) | 86 (42) | >2.4 (P) | >70 | - | No | Good | Death (after) | N/A | N/A |
| 12. † | Elassy  (2014) | 35 | Stuck PV (pannus) | 22 | MVRR (m) | 127 (45) | >2.4 (P) | >70 | - | No | Death (POD1)  Hemodynamic deterioration > cardiac arrest | Death (after) | N/A | N/A |
| 13. | Elassy  (2014) | 37 | Stuck PV | 18 | MVRR (m) | 117 (60) | >2.4 (P) | >70 | - | No | Good | Death (after) | N/A | N/A |
| 14. | Elassy  (2014) | 26 | Stuck PV | 21 | MVRR (m) | 95 (49) | >2.4 (P) | >70 | - | Yes | Good | Good | Term | CS |
| 15. | Elassy  (2014) | 36 | Stuck PV | 15 | MVRR (m) | 110 (57) | >2.4 (P) | >70 | - | Yes | Good | Death (after) | N/A | N/A |
| 16. | Elassy  (2014) | 34 | Stuck PV | 23 | MVRR (m) | 118 (65) | >2.4 (P) | >70 | - | Yes | Good | Death (after) | N/A | N/A |
| 17. | Elsayed  (2019) | 34 | Acute malfunction PV | 11 | MVRR (m) | - | - | - | - | No | Good | Death (after) | N/A | N/A |
| 18. | Elsayed  (2019) | 23 | Acute malfunction PV | 9 | MVRR (m) | - | - | - | - | No | Good | Death (after) | N/A | N/A |
| 19. | Elsayed  (2019) | 14 | Acute malfunction PV | ? | MVRR (m) | - | - | - | - | No | Good | Good | - | CS |
| 20.  † | Elsayed  (2019) | 28 | Acute malfunction PV | 13 | MVRR (m) | - | - | - | - | No | Death (POD12)  CVA | Death (after) | N/A | N/A |
| 21.  † | Elsayed  (2019) | 31 | Acute malfunction PV | 10 | MVRR (m) | - | - | - | - | No | Death (during)  Died on table | Death (during) | N/A | N/A |
| 22. | Elsayed  (2019) | 24 | Acute malfunction PV | 16 | MVRR (m) | - | - | - | - | No | Good | Good | - | CS |
| 23. | Elsayed  (2019) | 27 | Acute malfunction PV | 12 | MVRR (m) | - | - | - | - | No | Good | Good | - | CS |
| 24. | Elsayed  (2019) | 24 | Acute malfunction PV | 17 | MVRR (m) | - | - | - | - | No | Good | Good | - | CS |
| 25.  † | Elsayed  (2019) | 18 | Acute malfunction PV | 11 | MVRR (m) | - | - | - | - | No | Death (POD1)  Low COP | Death (after) | N/A | N/A |
| 26. | Elsayed  (2019) | 22 | Acute malfunction PV | 14 | MVRR (m) | - | - | - | - | No | Good | Death (after) | N/A | N/A |
| 27.  † | Elsayed  (2019) | 34 | Acute malfunction PV | 12 | MVRR (m) | - | - | - | - | No | Death (during)  Died on table | Death (during) | N/A | N/A |
| 28. | Elsayed  (2019) | 30 | Acute malfunction PV | 22 | MVRR (m) | - | - | - | - | No | Good | Death (after) | N/A | N/A |
| 29. | Elsayed  (2019) | 26 | Acute malfunction PV | 13 | MVRR (m) | - | - | - | - | No | Good | Good | - | CS |
| 30.  † | Elsayed  (2019) | 37 | Acute malfunction PV | 14 | MVRR (m) | - | - | - | - | No | Death (during)  Died on table | Death (during) | N/A | N/A |
| 31. | Foolchand  (2021) | 30 | Stuck PV | 15 | MVRR (m) | - | - | - | - | No | Good | Good | - | VD |
| 32. | Foolchand  (2021) | 20 | PVT (tPA-) | 21 | MVRR (m) | - | - | - | - | No | Good | Good | - | VD |
| 33. | Foolchand  (2021) | 19 | PVT (tPA-), AS | 18 | MVRR (m) + AVR (m) | - | - | - | - | No | Good | Death (after) | N/A | N/A |
| 34. | Foolchand  (2021) | 27 | PVT (tPA-) | 29 | MVRR (m) | - | - | - | - | No | Good | Death (after) | N/A | N/A |
| 35. | Golden  (1996) | 25 | RHD, AR | 17 | AVR (t) | 67 (53) | P | 58-78 | 32 | No | Pericardial effusion > corticosteroids  Good | Good | 40 | VD |
| 36. | Gupta  (2019) | 37 | PVT (tPA-) | 9 | MVRR (t) | - | - | - | - | No | Good | Death (after) | N/A | N/A |
| 37. | Iscan  (2006) | 28 | PVT (tPA-) | 18 | MVRR (t) | - | HF (NP) | >60 | 33 | Yes | Good | Good | - | CS |
| 38. | Iscan  (2006) | 25 | PVT (tPA-) | 14 | MVRR (m) | 75 (36) | HF (NP) | >60 | 33 | Yes | Good | Good | - | CS |
| 39. | Izquierdo  (1988) | 33 | PVT (tPA-) | 29 | MVRR (m) | - | HF | HP | 33 | Yes | Good | Death (during) | N/A | N/A |
| 40. | Jadhav  (2023) | 22 | PVT (tPA+) | 14 | MVRR (m) | - | - | - | - | No | Good | Good | 38 | CS |
| 41. | Jafferani  (2010) | 39 | MS | 22 | MVR (m) | 90 (70) | - | - | >32 | No | CS wound hematoma > reoperation | Good, twins | 33 | CS |
| 42. | Jafferani  (2010) | 25 | AS | 25 | AVR (m) | 150 (101) |  |  | >32 | No | Good | FGR | 34 | CS |
| 43. | James  (1998) | 30 | PVT (tPA-) | 8 | AVRR (m) | - | - | - | - | No | Cardiac arrest, resuscitation, anoxic encephalopathy | Death (after) | N/A | N/A |
| 44. | Johnston  (2014) | 34 | PV mismatch, AS | 20 | AVRR (m) | - | - | - | - | No | Good | Death (after) | N/A | N/A |
| 45. | Kaoutzanis  (2012) | 23 | IE, BAV, AR | 23 | AVR (t) | 95 (70) | >3.85 (P) | >70 | N | Yes, bradycardias | Good | Death (after) | N/A | N/A |
| 46. | Khan  (2003) | 31 | AR | 12 | AVR (t) |  | (P) | >65 | 35 | Yes, good | Good | Good | 38 | CS |
| 47. | Kikon  (2014) | 25 | RHD, MR | 28 | MVR (m) | 63 (34) | >2.5 (NP) | >70 | N | Yes, good | Good | Good | 38 | CS |
| 48. | Kisat  (2011) | 35 | MS | 22 | MVR (m) | 90 (70) | - | - | - | Yes, good | Good | Good, twins | 33 | CS |
| 49. | Kole  (1997) | 25 | Stuck PV (pannus) | 20 | MVRR (m) | 70 (35) | 2.5 | 65-70 | 28 | Yes, good | Good | Good | Term | CS |
| 50. | Korsten  (1989) | 36 | IE | 24 | AVR (m) | 114 (88) | HF (NP) | 75 | >35 | Yes, decelerations | CVA, right hemiparesis | Good | 37 | VD |
| 51. | Lin  (2008) | 34 | MS | 30 | MVRR (m) | 81 (33) | 2.4-2.6 (P) | - | N | Yes, bradycardias | Good | Good | 37 | CS |
| 52. | Marcoux  (2009) | 27 | IE, AR | 22 | AVR (t) | 78 (58) | 3.6-3.8 (P) | 65-75 | 36.5 | Yes, one bradycardia | Good | Good | 34 | CS |
| 53. | Masuda  (2019) | 22 | IE, MR | 24 | MVR (m) | 169 (128) | 120% of theoretical value | - | N | Yes | Good | Good | 38 | VD |
| 54. | Miller  (1978) | 35 | MS | 6 | MVR (m) | 45 (15) | - | 50 | 30 | No | MVRR before delivery due to obstructed MVP | Fetal distress, generalized hypertonicity | Term | VD |
| 55. | Mokgwathi  (2014) | 17 | MS, MR | 33 | MVR (?) | 50 (39) | 3.6-4.1 | 60-100 | 33-36 | Yes, bradycardia | Good | Fetal distress | 36 | CS |
| 56. | Mooij  (1988) | 36 | IE, AR | 24 | AVR (m) | 144 (99) | HF (NP) | 75 | N | Yes, decreased variability, decelerations | CVA | Good | 38 | VD |
| 57. | Mora  (1987) | 28 | AR, malfunction MVP | 30 | AVRR (m) + MVRR (m) | 160 | 52-64 ml/kg/min (NP) | 55-60 | 25 | No | Good | Fetal bradycardia after surgery, normalized after tocoylysis. Good | 33 | CS |
| 58. | Motherwell  (2006) | 20 | PVT (tPA-) | 32 | AVRR (?) | - | - | - | - | No | Good | Death (after) | N/A | N/A |
| 59. | Munoz-Mendoza  (2016) | 21 | PV malfunction | 16 | MVRR (m) | 145 | 4-5 L/min | 70 | - | Yes, fetal demise | Good | Death (during) | N/A | N/A |
| 60. | Muretti  (2010) | 24 | IE, MR | 22 | MVR (t) | 63 (47) | 3.0 (P) | 70 | 35 | Yes, good | Good | Good | 34 | CS |
| 61. | O’Donnell  (1983) | 21 | RHD | 31 | AVR (t) + MVR (t) | - | - | - | - | No | Good | Good | 32 | VD |
| 62. | Paulus  (1994) | 24 | IE, AR | 15 | AVR (t) | 150 | 61 ml/kg/min | 60-80 | 28 | Yes | Good | Death (after, POD1) | N/A | N/A |
| 63. | Pitfield  (2023) | 31 | IE, AR | 15 | AVR (m) | 154 (104) | 2.8 (P) | 50-55 | 36-37 | No | Threatened abortion POD1 | Good | 37 | CS |
| 64. | Price  (2009) | 36 | IE, TR | 19 | TVR (t) | 102 (78) | >3.0 (P) | 69-81 | 36-37 | No | Good | Death (POD10) | N/A | N/A |
| 65. | Sathananthan  (2019) | 29 | PVT (tPA-) | 8 | TVRR (t) | 96 | 2.6-3.0 (P) | - | 36-37 | Yes | Good | Good | 39 | VD |
| 66. | Siddiqui  (2008) | 25 | AS | 20 | AVR (?) | - | 2.5 L/min (NP) | 50-60 | 30 | No | Good | Good | 37 | CS |
| 67. | Sutton  (2005) | 26 | MR | 26 | MVR (m) | 99 (83) | 2.7 (P) | >70 | 35-38 | Yes | Complete heart block > PM | Death (PP day 6, CVA) | 27 | CS |
| 68. | Tehrani  (2004) | 23 | PVT (tPA-) | 27 | AVRR (m) | - | >2.5 | 70 | N | Yes, good | Mediastinal bleeding | Good | 38 | CS |
| 69. | Tripp  (1999) | 25 | AR | 14 | AVR (t) | 67 (53) | >2.5 (P) | 68-74 | 32 | Yes, good | Good | Good | 39 | - |
| 70. | Van Steenbergen  (2020) | 29 | PVT (tPA-) | 12 | AVRR (m) | - | - | - | - | No | Good | Good | 38 | CS |
| 71. | Vosa  (1988) | 21 | PVT (tPA-) | 11 | MVRR (m) | - | - | - | 28 | No | Good | Good | 35 | CS |
| 72. | Vosa  (1988) | 36 | RHD | 9 | MVR (m) | - | - | - | 28 | No | Good | Good | 37 | - |
| 73. | Vrkocova  (2016) | 29 | PVT (tPA+) | 6 | MVRR (t) | - | - | - | - | No | AV-block > PM | Death (after, therapeutic abortion) | N/A | N/A |
| 74. | Zellner  (1998) | 24 | PVT (tPA-) | 12 | MVRR (m) | 152 (104) | - | - | - | No | Good | Good | - | - |

† Maternal death.

Abbreviations: AR, aortic regurgitation; AS, aortic stenosis; AVR, aortic valve replacement; AVRR, aortic valve re-replacement; AV, atrioventricular; BAV, bicuspid aortic valve; COP, cardiorespiratory optimal point; CS, caesarean section; CVA, cerebrovascular accident; FGR, fetal growth restriction; H, hyperthermia; HELLP, hemolysis, elevated liver enzymes and low platelets; IE, infective endocarditis; LMWH, low molecular weight heparin; m, mechanical prosthesis; MR, mitral regurgitation; MS, mitral stenosis; MVP, mitral valve prosthesis; MVR, mitral valve replacement; MVRR, mitral valve re-replacement; N, normothermia; N/A, not applicable; NP, non-pulsatile; P, pulsatile; PM, pacemaker; POD, postoperative day; PP, postpartum; PV, prosthetic valve; PVT, prosthetic valve thrombosis; RHD, rheumatic heart disease; t, bioprosthesis; tPA, tissue plasminogen activator; TR, tricuspid regurgitation; TVR, tricuspid valve replacement; TVRR, tricuspid valve re-replacement; UFH, unfractionated heparin; VD, vaginal delivery; VKA, vitamin K antagonist.

**Supplementary Table 5.** Clinical features at presentation.

| Case no. | First author (year) | Clinical features |
| --- | --- | --- |
| 1. | Bahary  (1980) | Class III RHD with severe mitral stenosis. |
| 2. | Botta  (2015) | Persistent fever, child, dyspnea for mild efforts. TTE: severe mitral regurgitation, chordal ruptures, leaflet perforations, multiple vegetations, increased end-diastolic diameter (56mm) and volume (119ml). |
| 3. | Boulemden  (2018) | NYHA class III, no improvement after percutaneous balloon mitral valvuloplasty. |
| 4. | Carnero  (2009) | Congestive cardiac failure, oligoanuria, acidosis. TTE: prosthetic thrombosis, severe stenosis, moderate regurgitation. |
| 5. | Da Cunha  (2007) | NYHA class IV heart failure, pulmonary edema. TTE: mitral valve area 1.0cm^2^, left atrial thrombus. |
| 6. | Deviri  (1984) | Pulmonary edema, dyspneic after mild exertion. TTE: severe mitral prosthetic valve stenosis. |
| 7. | Dubrey  (2010) | Exertional breathlessness, wheezing, pulmonary edema despite maximal medical therapy. TTE: mitral valve area 1.0cm^2^, mean pulmonary artery pressure 87mmHg. |
| 8. | Eilen  (1981) | Recurrent peripheral finger embolizations without evidence of congestive heart failure. TTE: large multiple aortic valve vegetations. |
| 9. | Elassy  (2014) | Unknown |
| 10. | Elassy  (2014) | Unknown |
| 11. | Elassy  (2014) | Unknown |
| 12. | Elassy  (2014) | Unknown |
| 13. | Elassy  (2014) | Unknown |
| 14. | Elassy  (2014) | Unknown |
| 15. | Elassy  (2014) | Unknown |
| 16. | Elassy  (2014) | Unknown |
| 17. | Elsayed  (2019) | Unknown |
| 18. | Elsayed  (2019) | Unknown |
| 19. | Elsayed  (2019) | Unknown |
| 20. | Elsayed  (2019) | Unknown |
| 21. | Elsayed  (2019) | Unknown |
| 22. | Elsayed  (2019) | Unknown |
| 23. | Elsayed  (2019) | Unknown |
| 24. | Elsayed  (2019) | Unknown |
| 25. | Elsayed  (2019) | Unknown |
| 26. | Elsayed  (2019) | Unknown |
| 27. | Elsayed  (2019) | Unknown |
| 28. | Elsayed  (2019) | Unknown |
| 29. | Elsayed  (2019) | Unknown |
| 30. | Elsayed  (2019) | Unknown |
| 31. | Foolchand  (2021) | TTE (routine): dysfunction of single mitral leaflet. |
| 32. | Foolchand  (2021) | TTE: one stuck leaflet. |
| 33. | Foolchand  (2021) | Unknown |
| 34. | Foolchand  (2021) | Asymptomatic. TTE: stuck mitral valve leaflet. |
| 35. | Golden  (1996) | New onset mild angina unrelated to exertion, dyspnea on exertion. Echocardiogram: moderate to severe aortic regurgitation. MRI: severe aortic regurgitation, ascending aortic aneurysm, dilated left ventricle with globally diminished function (40%). |
| 36. | Gupta  (2019) | Dyspnea with NYHA class IV symptoms, mild bibasilar rales, no jugular venous distension or pedal edema. TTE: normal left ventricular ejection fraction (70%), dilated left atrium, mean transmitral diastolic gradient of 23mmHg (HR 98bpm), peak mitral diastolic velocity 266cm/sec, mitral pressure half time 235ms. TEE: large thrombus 3-4cm^2^ |
| 37. | Iscan  (2006) | Unknown |
| 38. | Iscan  (2006) | Unknown |
| 39. | Izquierdo  (1988) | Pulmonary edema. TTE: mitral valve area <0.7cm. |
| 40. | Jadhav  (2023) | Sudden onset dyspnea, palpitation, chest discomfort. HR 134bpm, BP 82/50mmHg, respiratory rate 56/min, oxygen saturation 72%, crepitations across all lung fields, loss of metallic click sound. TTE: stuck mitral prosthetic valve, velocity 2.3m/s, peak gradient 23mmHg, mean gradient 16mmHg, thrombus noted attached to valve leaflet, normal left ventricular function, right ventricular systolic pressure 48mmHg. No improvement after thrombolysis. |
| 41. | Jafferani  (2010) | Shortness of breath, NYHA class III, BP 90/60mmHg, HR 90bpm, respiratory rate 20/min, oxygen saturation 96%. TEE: mitral valve area 0.7cm, peak pressure gradient 39mmHg, mean gradient 28mmHg, severely dilated left atrium, thrombus in left atrial appendage, another thrombus protruding into the left atrium with pulmonary outflow obstruction. |
| 42. | Jafferani  (2010) | NYHA class III, BP 100/70mmHg, HR 95bpm, respiratory rate 22/min, oxygen saturation 95%. Echocardiogram: calcified aortic valve with severely restricted cusp mobility, peak gradient 150mmHg, mean gradient 80mmHg, pulmonary artery systolic pressure 20mmHg, aortic valve area 0.5cm^2^. |
| 43. | James  (1998) | Unknown |
| 44. | Johnston  (2014) | NYHA class I. Echocardiogram: aortic valve area 0.27cm^2^, peak gradient 86mmHg, ejection fraction 65-70%. Fluoroscopy: normal functioning valve without vegetation or thromboses. TEE: indexed effective orifice area 0.4cm^2^, peak gradient 120mmHg. |
| 45. | Kaoutzanis  (2012) | Unknown |
| 46. | Khan  (2003) | Palpitations, met criteria for Marfan’s syndrome. TEE: aortic regurgitation with aortic root of 6.9cm in diameter. |
| 47. | Kikon  (2014) | NYHA class III-IV, massive hemoptysis, BP 88/64mmHg, HR 98bpm. Chest x-ray: cardiomegaly with pulmonary plethora. TTE: mitral valve area 0.9cm^2^, mean gradient 33mmHg, vena contracta 0.7cm, severe pulmonary artery hypertension, left atrial size 7.4cm, dilated left ventricle (end systolic dimension 50mm). Patient status was deteriorated despite all conservative measures. |
| 48. | Kisat  (2011) | NYHA class III-IV, BP 106/73mmHg, HR 100bpm, oxygen saturation 96%, jugular venous distension. Echocardiogram: mitral valve area 0.6cm^2^, mean gradient 20mmHg, pulmonary artery pressure 80mmHg. |
| 49. | Kole  (1997) | Acute pulmonary edema and cardiogenic shock, absent valve click. Echocardiography: stuck mitral disc prosthesis in open position, mean gradient 23.5mmHg, moderate pulmonary hypertension. Fast deterioration. |
| 50. | Korsten  (1989) | Unknown |
| 51. | Lin  (2008) | Unknown |
| 52. | Marcoux  (2009) | Septic shock and congestive heart failure, multiple cerebral and peripheral embolic events. TEE: severe aortic regurgitation. |
| 53. | Masuda  (2019) | Congestive heart failure, dyspnea despite only mild effort, persistent fever, chills. Chest x-ray: butterfly shadow. TTE: severe mitral valve regurgitation with mobile vegetation (20mm in diameter). Progression to massive regurgitation and hemodynamic instability. |
| 54. | Miller  (1978) | Cardiac failure. |
| 55. | Mokgwathi  (2014) | NYHA class III, WHO heart failure stage C. BP 90/60mmHg, HR 82bpm. Echocardiogram: mitral valve area 0.88cm^2^, mean gradient 1.85m/s or 15mmHg, right ventricular pressure 42mmHg, ejection fraction 52%. |
| 56. | Mooij  (1988) | Left heart failure, right-sided hemiparesis. Echocardiogram: severe aortic regurgitation. |
| 57. | Mora  (1987) | Unknown |
| 58. | Motherwell  (2006) | Syncope, dyspnea, chest pain, pulmonary edema. Echocardiogram: left ventricular hypertrophy, reduced aortic valve opening and annular thrombus, velocity 8m/s, pressure gradient 240mmHg. |
| 59. | Munoz-Mendoza  (2016) | Decompensated heart failure, NYHA class III. TTE: severe mitral stenosis with thickened prosthetic leaflets, mean pressure gradient 44Mhg, right ventricular systolic pressure 85mmHg. TEE: no thrombus nor vegetation was seen. |
| 60. | Muretti  (2010) | Unknown |
| 61. | O’Donnell  (1983) | Three peripheral embolic episodes, vegetations and precarious hemodynamic state with persistent tachycardia. |
| 62. | Paulus  (1994) | Congestive heart failure, anemia, mild renal failure with hematuria. Chest x-ray: small bilateral pleural effusion, possible left lower lobe infiltrate. Echocardiogram: large area of increased density at the right coronary cusp of the aortic valve. Deterioration of status: agitated and confused. |
| 63. | Pitfield  (2023) | Tachycardia and fatigue. Echocardiogram: severe aortic regurgitation, multiple aortic valve vegetations (largest 37x5mm), extensive destruction of aortic valve, small aortic root abscess, left ventricular dilation, depressed left ventricular systolic function (20-25%). |
| 64. | Price  (2009) | Hemodynamic instability and failure of medical therapy during a two-week stay in the ICU. |
| 65. | Sathananthan  (2019) | Unknown |
| 66. | Siddiqui  (2008) | Grade III dyspnea. Echocardiogram: calcified aortic valve with ventricular movement, mild symptomatic hypertrophied normal sized left ventricle. |
| 67. | Sutton  (2005) | Shortness of breath, thromboembolism, splenic infarct, renal infarct, atrial fibrillation. TTE: myxomatous mitral valve with a flail anterior leaflet associated with chordal rupture, severe mitral regurgitation with posterior jet, attached to the flail mitral leaflet were small echogenic masses suspicious for thrombi or vegetative colonies. |
| 68. | Tehrani  (2004) | Acute left heart failure. TTE: minimal leaflet excursion with evidence of valvular thrombus. |
| 69. | Tripp  (1999) | Class III dyspnea on exertion. Echocardiogram: dilated aortic root (6.8cm in diameter), moderate to severe aortic regurgitation, mild left ventricular hypertrophy. |
| 70. | Van Steenbergen  (2020) | NYHA class III. TTE: severe aortic regurgitation with aortic backflow, impaired opening of the valve with preserved systolic ventricular function. Progressive valve dysfunction despite adequate anticoagulant therapy. |
| 71. | Vosa  (1988) | Dyspnea, absence of prosthetic closure sound. Echocardiogram: reduction motion of disk. |
| 72. | Vosa  (1988) | Pulmonary congestion. Deterioration despite intensive medical care. |
| 73. | Vrkocova  (2016) | Unknown |
| 74. | Zellner  (1998) | Shortness of breath, hemoptysis. Echocardiogram: compromised valve leaflet movement. |

**Supplementary Table 6.** Extent of missing data in numbers (%)

| **Variable** | **Number of cases with missing data (%)** |
| --- | --- |
| Maternal age during pregnancy, years | 2 (2.4) |
| Gestational age during pregnancy, weeks | 1 (1.2) |
| Indication for valve replacement | 0 |
| Type of valve | 4 (4.7) |
| Position of valve | 0 |
| Re-replacement | 0 |
| CBP duration, min | 44 (51.8) |
| Cross-clamp time, min | 50 (58.8) |
| Hypothermia | 47 (55.3) |
| Pulsatile flow | 57 (68.2) |
| Pump flow, L/min/m^2^ | 63 (74.1) |
| Mean arterial pressure, mmHg | 52 (61.2) |
| Fetal monitoring during surgery | 0 |
| Maternal death | 0 |
| Maternal complications | 0 |
| Fetal death | 0 |
| Delivery | 2 (2.4) |
| Gestational age during delivery, weeks | 13 (24.1) |
| Preterm birth | 13 (24.1) |
| Caesarean section | 2 (3.7) |

**Supplementary Table 7.** Surgery characteristics and outcomes stratified by LMIC or HIC

|  | LMIC  (n=47) | HIC  (n=38) | P-value |
| --- | --- | --- | --- |
| Maternal age during pregnancy (years) | 27.1 ± 5.9 | 28.8 ± 6.0 | 0.189 |
| Gestational age during valve replacement | 18.2 ± 6.6 | 19.2 ± 7.9 | 0.534 |
| Trimester |  |  | 0.444^#^ |
| 1^st^ trimester | 16 (34.8) | 13 (34.2) |  |
| 2^nd^ trimester | 26 (56.5) | 18 (47.4) |  |
| 3^rd^ trimester | 4 (8.7) | 7 (18.4) |  |
|  |  |  |  |
| Indication for valve replacement |  |  | **<0.001**^#^ |
| Valve thrombosis | 7 (14.9) | 14 (36.8) | **0.024** |
| Stuck PV / acute malfunction PV | 22 (46.8) | 5 (13.2) | **0.001** |
| Endocarditis | 0 (0) | 10 (26.3) | **<0.001** |
| Native valve disease** | 18 (38.3) | 9 (23.7) | 0.168 |
|  |  |  |  |
| Type of valve |  |  |  |
| Mechanical valve | 41 (91.1) | 24 (66.7) | **0.010** |
| Tissue valve | 4 (8.9) | 12 (33.3) | **0.010** |
|  |  |  |  |
| Position |  |  |  |
| Aortic | 7 (14.9) | 15 (39.5) | **0.013** |
| Mitral | 36 (76.6) | 20 (52.6) | **0.024** |
| Aortic + Mitral | 3 (8.5) | 1 (2.6) | 0.374 |
| Tricuspid | 0 (0) | 2 (5.3) | 0.197 |
|  |  |  |  |
| Re-replacement | 29 (61.7) | 19 (50.0) | 0.379 |
|  |  |  |  |
| CPB duration (min) | 95 ± 30 | 106 ± 40 | 0.240 |
| Cross-clamp time (min) | 59 ± 28 | 73 ± 28 | 0.149 |
| Hypothermia | 13 (92.9) | 13 (54.2) | **0.027** |
| Pulsatile flow | 8 (66.7) | 12 (80.0) | 0.662 |
| Pump flow (L/min/m^2^) | 2.5 ± 0.4 | 3.0 ± 0.6 | **0.037** |
| Mean arterial pressure (mmHg) | 67 ± 7 | 69 ± 6 | 0.378 |
| Fetal monitoring during surgery | 9 (19.1) | 18 (47.4) | **0.009** |
|  |  |  |  |
| Maternal death | 7 (14.9) | 1 (2.6) | 0.070 |
| Maternal complications*** | 2 (5.0) | 10 (27.0) | **0.011** |
| Fetal death | 18 (38.3) | 11 (28.9) | 0.491 |
|  |  |  |  |
| Delivery | 29 (61.7) | 25 (69.4) | 0.495 |
| Gestational age during delivery (weeks) | 36.1 ± 2.6 | 36.7 ± 3.5 | 0.546 |
| Preterm birth | 7 (43.8) | 6 (24.0) | 0.302 |
| Caesarean section | 24 (82.8) | 13 (56.6) | 0.063 |

**Aortic stenosis and/or regurgitation or mitral stenosis and/or regurgitation in the context of rheumatic heart disease (n=12), congenital heart disease (n=4) or unknown etiology (n=11).

***Maternal death cases excluded.

^#^P-value for test of joint significance.

Data are presented as n (%) or mean ± SD unless otherwise specified. Percentages are calculated using pairwise deletion.

Abbreviations: CPB, cardiopulmonary bypass; HIC, high-income country; LMIC, low-or-middle-income country; PV, prosthetic valve.
